# Loss of function of Arabidopsis microRNA-machinery genes impairs fertility, and has effects on homologous recombination and meiotic chromatin dynamics

**DOI:** 10.1038/s41598-017-07702-x

**Published:** 2017-08-24

**Authors:** Cecilia Oliver, Mónica Pradillo, Sara Jover-Gil, Nieves Cuñado, María Rosa Ponce, Juan Luis Santos

**Affiliations:** 1 Departamento de Genética, Facultad de Biología, Universidad Complutense de Madrid, 28040 Madrid, Spain; 2 Instituto de Bioingeniería, Universidad Miguel Hernández, Campus de Elche, 03202 Elche, Spain; 3 Present Address: Institut de Génétique Humaine UMR9002 CNRS-Université de Montpellier, 34396 Montpellier, cedex 05, France

## Abstract

MicroRNAs (miRNAs) are ~22-nt single-stranded noncoding RNAs with regulatory roles in a wide range of cellular functions by repressing eukaryotic gene expression at a post-transcriptional level. Here, we analyzed the effects on meiosis and fertility of hypomorphic or null alleles of the *HYL1*, *HEN1*, *DCL1*, *HST* and *AGO1* genes, which encode miRNA-machinery components in Arabidopsis. Reduced pollen and megaspore mother cell number and fertility were shown by the mutants analyzed. These mutants also exhibited a relaxed chromatin conformation in male meiocytes at the first meiotic division, and increased chiasma frequency, which is likely to be due to increased levels of mRNAs from key genes involved in homologous recombination. The *hen1*-*13* mutant was found to be hypersensitive to gamma irradiation, which mainly causes double-strand breaks susceptible to be repaired by homologous recombination. Our findings uncover a role for miRNA-machinery components in Arabidopsis meiosis, as well as in the repression of key genes required for homologous recombination. These genes seem to be indirect miRNA targets.

## Introduction

MicroRNAs (miRNAs) are small (20–22 nt), single-stranded, non-coding RNAs encoded by endogenous loci: the *MIR* genes. In Arabidopsis, transcription of *MIR* genes by RNA polymerase II yields primary miRNA transcripts (pri-miRNAs) that fold into hairpin structures. The type III endoribonuclease DICER-LIKE-1 (DCL1), in coordination with the RNA-binding proteins SERRATE (SE), TOUGH (TGH) and HYPONASTIC LEAVES 1 (HYL1), binds and processes pri-miRNAs into miRNA precursors (pre-miRNAs). These pre-miRNAs are then cleaved into miRNA:miRNA* duplexes, which are stabilized by methylation at their 3′ ends by HUA ENHANCER 1 (HEN1). The HASTY (HST) exportin is thought to be required for nuclear export of miRNAs in Arabidopsis. Once in the cytoplasm, the miRNA strand of the miRNA:miRNA* duplex is loaded onto the RNA-induced silencing complex (RISC), which contains an ARGONAUTE (AGO) protein. Complementary base pairing allows miRNAs to select their targets, and the ribonuclease AGO1 carries out gene silencing by slicing mRNAs or attenuating their translation^1^. AGO1 has also been detected in the nucleus, suggesting an alternative nuclear AGO1-RISC assembly^2, 3^.

Meiosis is a specialized cell division that yields new allele combinations in the gametes; it is essential for maintaining chromosome number across generations. Although miRNAs are known to regulate many aspects of plant growth and development, as well as hormonal and stress responses^4^, our understanding of their role in meiosis is very limited. Two miRNA families are required for sperm production in the male germline of mammals^5^, and distinct miRNAs are down- or up-regulated during reproductive development in plants^6^. Next generation sequencing identified 33 miRNAs in the Arabidopsis male gametophyte^7, 8^. Additionally, the so-called phased secondary siRNAs (phasiRNAs)—whose function and target genes remain elusive—are abundant during male gametogenesis in plants^1, 9^.

Several lines of evidence indicate a role for plant AGO proteins in meiosis. In rice, MEIOSIS ARRESTED AT LEPTOTENE 1 (MEL1), a core component of the male germline-specific RISC, is required for pollen grain development^10, 11^. AGO104, the maize ortholog of Arabidopsis AGO9, is involved in female meiosis^12^. Mutation of Arabidopsis *AGO4*, *AGO6*, *AGO8* and *AGO9* lead to abnormal female gametophyte precursors^13, 14^. In addition, the Arabidopsis *ago2*-*1* mutant exhibits increased cell chiasma frequency in pollen mother cells (PMCs). Chiasma frequency and fertility are normal in Arabidopsis *ago9*-*1*, which displays a high frequency of chromosome interlocks from pachytene to metaphase^15^.

To gain insight on the role of the miRNA machinery in Arabidopsis meiosis, we analyzed the effects of hypomorphic or null alleles of *AGO1*, *HYL1*, *HEN1*, *DCL1* and *HST* on fertility and meiosis. Mutations in these genes impair processes regulated by miRNAs, causing derepression of their target genes. Mutations in *HEN1*, *AGO1* and *DCL1* also alter pathways guided by other small RNAs^16, 17^. The mutants examined here share several meiotic phenotypes: decreased number of cells that enter meiosis, increased number of chiasmata, and partial chromosome decondensation from pachytene to metaphase I. These phenotypes could be associated with changes in the expression of genes involved in chromatin remodeling and homologous recombination (HR) in gamete-containing tissues. Interestingly, changes in the expression profiles of these genes are also found in somatic tissues from these mutants. Our results uncover a role for the miRNA pathway in the regulation of meiotic chromatin organization and HR.

## Results

### Fertility is impaired by mutations in miRNA-machinery genes

In Arabidopsis, loss-of-function of miRNA-machinery genes severely reduces fertility, leading to complete sterility or early lethality^18^. To investigate the causes of such sterility, we characterized meiosis in mutants carrying partial or complete loss-of-function alleles of genes involved in different steps of the miRNA pathway: *DCL1* and *HYL1*, in miRNA precursor processing; *HEN1*, in miRNA:miRNA* duplex stabilization; *HST*, in nuclear export of miRNAs; and *AGO1*, in silencing of miRNA targets. The studied mutants were in the Col-0 and L*er* genetic backgrounds, and carry different types of lesions (see Methods and Supplementary Table S1). Since null alleles of *AGO1* and *DCL1* cause early lethality precluding the study of meiosis, we used their hypomorphic and viable *ago1*-*52* ^18^ and *dcl1*-*9* ^19^ alleles. The alleles of *HYL1*, *HEN1* and *HST* studied here are assumed to be null^18^.

To estimate the extent of fertility in the mutants under study, we determined the number of seeds per silique. Only *dcl1*-*9* and *ago1*-*101* were completely sterile, while the remaining mutants displayed short siliques and a variable degree of semi-sterility with significant decreases in the number of seeds per silique (8.98 ± 0.98 in *hen1*-*6*, 9.00 ± 0.42 in *hyl1*-*12*, 7.50 ± 0.49 in *hst*-*21*, and 7.00 ± 1.78 in *ago1*-*52*) compared to their corresponding wild-types (44.5 ± 2.11 in L*er* and 45.95 ± 3.60 in Col-0) (Supplementary Fig. S1).

**Figure 1:**
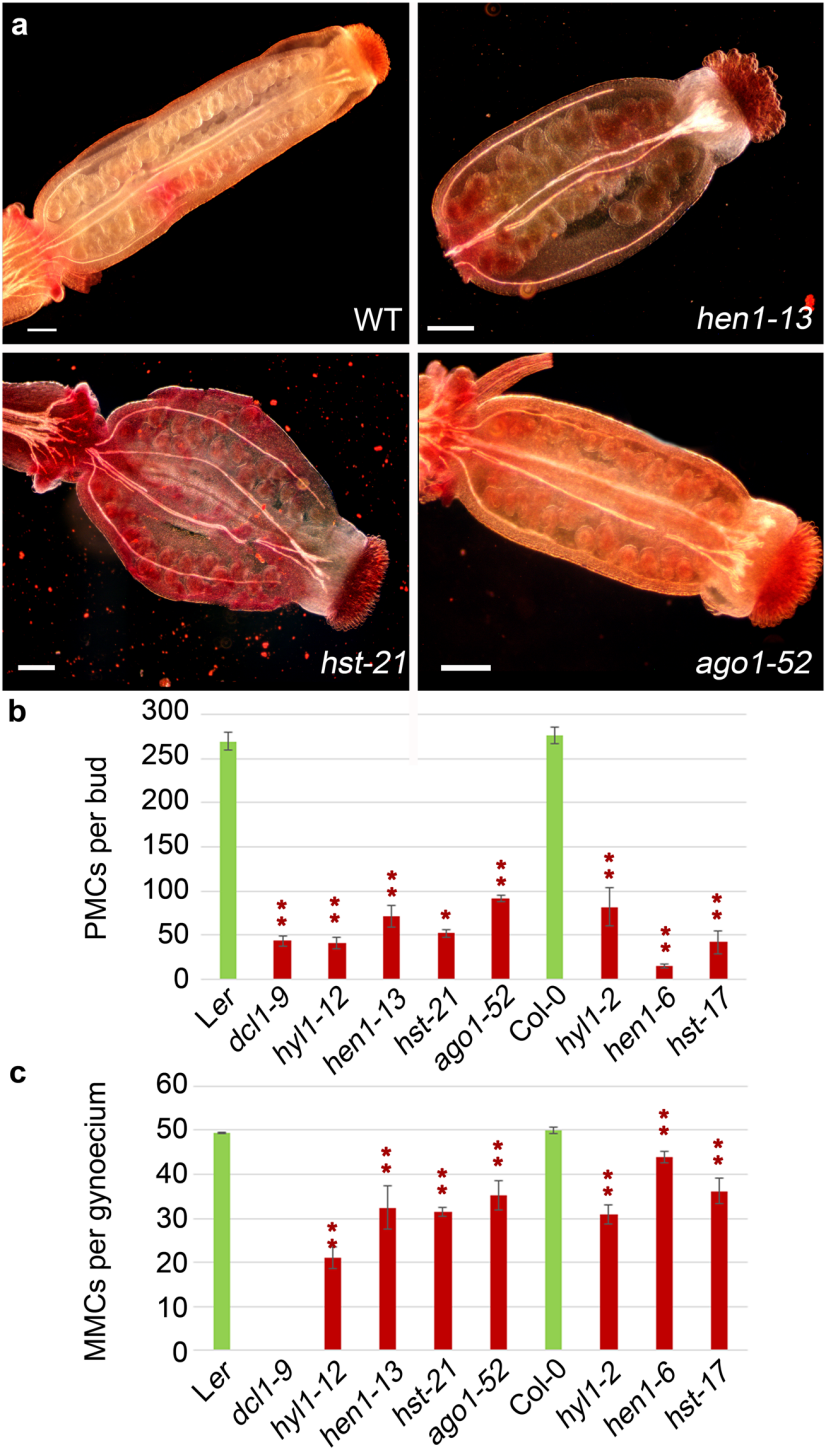
Gynoecium morphology, and PMC and MMC number in the *hen1*-*13*, *hst*-*21* and *ago1*-*52* mutants. (**a**) Representative images of carmine staining of gynoecia from L*er* and the *hen1*-*13*, *hst*-*21* and *ago1*-*52* mutants. (**b**) PMC number per bud. (**c**) MMC number per gynoecium. Asterisks indicate a significant difference with the corresponding wild type in a Mann-Whitney U test (*p < 0.05 and **p < 0.01).

Since developmental alterations in flower organs and/or a reduction in the production of viable gametes might account for the observed decrease in fertility, we analyzed the number of PMCs per bud, and megaspore mother cells (MMCs) per gynoecium. The mutants displayed a reduction in PMCs ranging from 3-fold (in *ago1*-*52*) to 20-fold (in *hen1*-*6*) compared to their wild types (Fig. 1b). The reduction in MMC number was less severe, except for *hyl1*-*12*, which exhibited less than half of MMCs per gynoecium (21.00 ± 2.40) than Col-0 (49.40 ± 0.24; Fig. 1a,c). Our results agree with previous studies highlighting the poor transmission of the *ago1*-*10* hypomorphic and *hyl1*-*1* null alleles through male gametes^20^.

Only the completely sterile *ago1*-*101* and *dcl1*-*9* mutants displayed abnormally shaped reproductive organs. Neither ovules (embryonic sacs) in the gynoecium nor PMCs in the anthers were observed in *ago1*-*101* (Supplementary Fig. S2). Flowers with altered organ numbers (two gynoecia, three petals and three sepals) were occasionally seen in *ago1*-*101*. *dcl1*-*9* showed a variable number of carpels, which sometimes fused to form a gynoecium, and numerous anthers (6 to 28) in different developmental stages. Pollen sacs occasionally embedded into carpel tissue were also observed (Supplementary Fig. S2). Aberrations in flower development have been previously reported for *dcl1* mutants (initially named *carpel factory*)^19^.

### Chromatin is decondensed at pachytene in most miRNA-machinery mutants

In Arabidopsis PMCs, the ten chromosomes appear as thread-like structures at leptotene, homologous synapsis starts at zygotene, and full synapsis is achieved at pachytene. Five bivalents are clearly observed after the disassembly of the synaptonemal complex (SC) at diplotene. Bivalents align at metaphase I plate and homologous chromosomes segregate to opposite poles at anaphase I. Four nuclei, each with a set of five chromatids, are formed during the second meiotic division.

**Figure 2:**
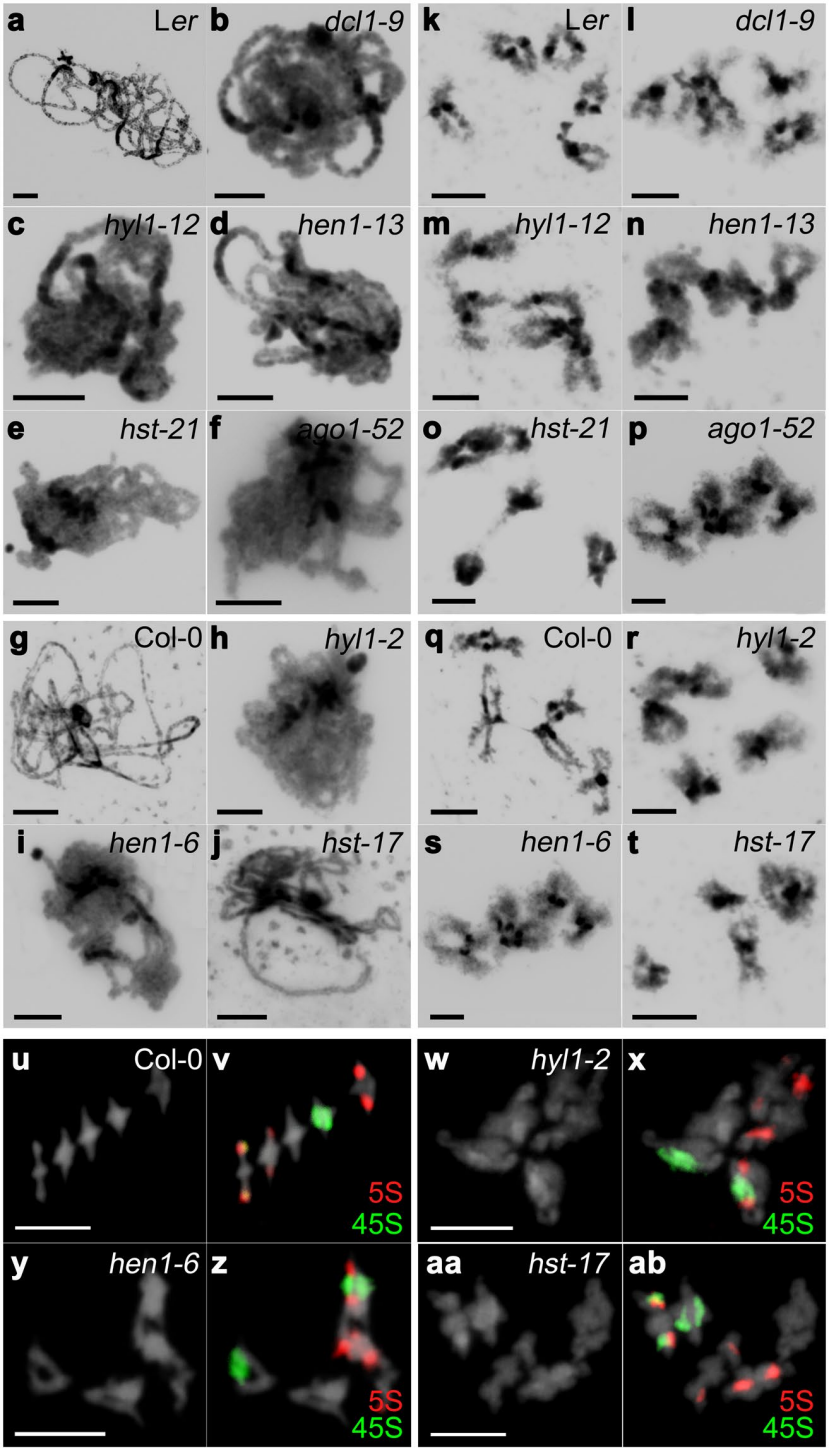
Representative images of DAPI stained PMCs at pachytene and diakinesis, and FISH of metaphases I. (**a**–**j**) Pachytene. (**k**–**t**) Diakinesis. (**u**–**ab**) Diakinesis-Metaphase I. (**a**,**k**) L*er*. (**b**,**l**) *dcl1*-*9*. (**c**,**m**) *hyl1*-*12*. (**d**,**n**) *hen1*-*13*. (**e**,**o**) *hst*-*21*. (**f**,**p**) *ago1*-*52*. (**g**,**q**,**u**,**v**) Col-0. (**h**,**r**,**w**,**x**) *hyl1*-*2*. (**i**,**s**,**y**,**z**) *hen1*-*6*. (**j**,**t**,**aa**,**ab**) *hst*-*17*. Mutant PMCs show a partial chromatin decondensation compared with wild type (see text for details). 5S rDNA is labeled in red and 45S rDNA in green. Bars = 5 µm.

All mutants, except *hst*-*17*, showed chromatin decondensation at pachytene (Fig. 2a–j), *i*.*e*., absence of the characteristic chromomeric pattern in the bivalents, and at diakinesis (Fig. 2k–t). The percentage of decondensed male meiocytes ranged from 26% in *hyl1*-*12* to 41% in *hen1*-*13* (Fig. 2k–t, Table S5). At metaphase I, decondensed bivalents were observed in *hen1*-*6*, *hst*-*17* and *hyl1*-*2* (13, 31 and 21% of cells, respectively) (Fig. 2u–ab, Supplementary Table S2). No differential cytological features among mutant and wild-type plants were observed from anaphase I onwards (Supplementary Fig. S3).

Because the mutants showed decondensation at pachytene, the synaptic process was studied by immunodetection of the ASYNAPSIS 1 (ASY1) and ZYPPER 1 (ZYP1) proteins, which are related to the SC. At zygotene/pachytene, ASY1 localizes to the base of chromatin loops, which are in close association with the axial/lateral elements of meiotic chromosomes. ZYP1 is detectable at zygotene as foci or very short stretches when progressive SC formation takes place. Continuous ZYP1 lines at pachytene indicate achievement of full synapsis. We did not observe differences in the localization patterns of ASY1 and ZYP1 among our mutants and wild-type plants, indicating that synapsis occurred normally (as example see Supplementary Fig. S4 displaying the immunolocalization signals on a *dcl1*-*9* PMC).

### Histone H3 epigenetic marks are not altered in miRNA-machinery mutants

**Figure 3:**
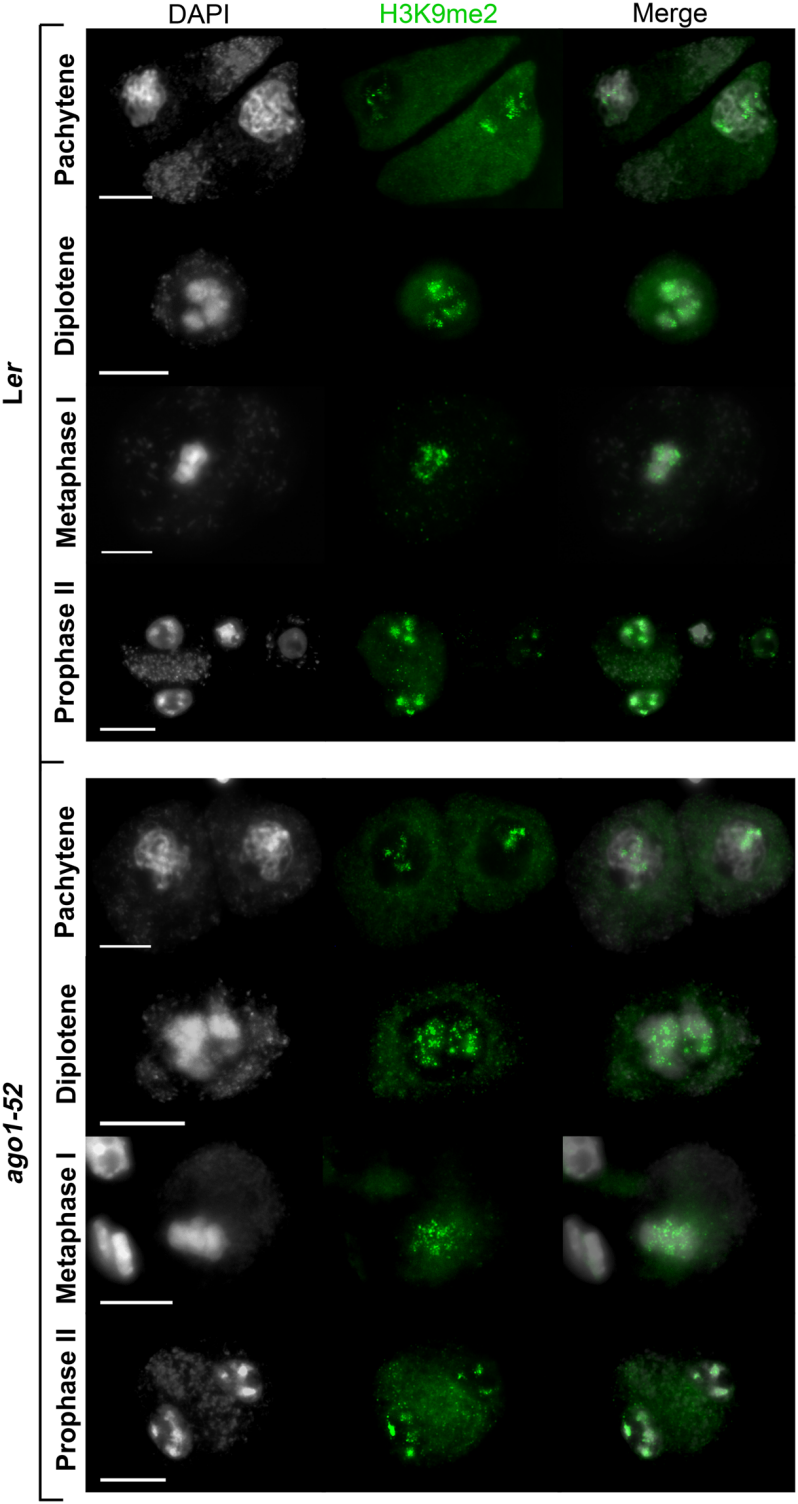
Distribution of H3K9me2 in PMCs from L*er* and *ago1*-*52*. Representative images of H3K9me2 immunolocalization, which marks constitutive heterochromatic regions. Similar results were obtained with the other mutants analyzed. Bars = 5 µm.

In Arabidopsis, dimethylation of lysine 9 (H3K9me2), which marks constitutive heterochromatin, is associated with condensation, while H3K4me2, H3K4me3, H3K27me3 and H3 acetylation (H3ac) specifically occur in decondensed euchromatin^21^. Serine 10 phosphorylation (H3S10Ph) specifically marks condensed chromatin. Chromosomes are only labeled with H3S10Ph at mitotic metaphase and from meiotic diplotene to metaphase I, and metaphase II. Different from other plant species, H3S10Ph marks are uniform along Arabidopsis chromosomes at these stages^22^. We analyzed the above-mentioned epigenetic marks in miRNA-machinery mutants, but the immunolabeling patterns observed were indistinguishable to those of their corresponding wild-type backgrounds. H3K9me2 was primarily restricted to the pericentromeric regions (Fig. 3). H3Ac immunosignals were invariably observed throughout meiosis (Supplementary Fig. S5). The immunolabeling patterns corresponding to H3K4me2, H3K4me3, and H3K27me3 were similar, being these modifications distributed across all chromosomes (Supplementary Figs S6–S8). We neither found differences of the chromosomal spatial and temporal distribution of H3S10Ph (Supplementary Fig. S9), since it appeared at late prophase I at the whole condensed chromosomes, disappearing at telophase I and reappearing at late prophase II. However, we cannot exclude specific differences affecting small chromosomal regions due to the limited resolution of the microscopic observations.

### Genes involved in chromatin structure maintenance or modification are up-regulated in miRNA-machinery mutants

**Figure 4:**
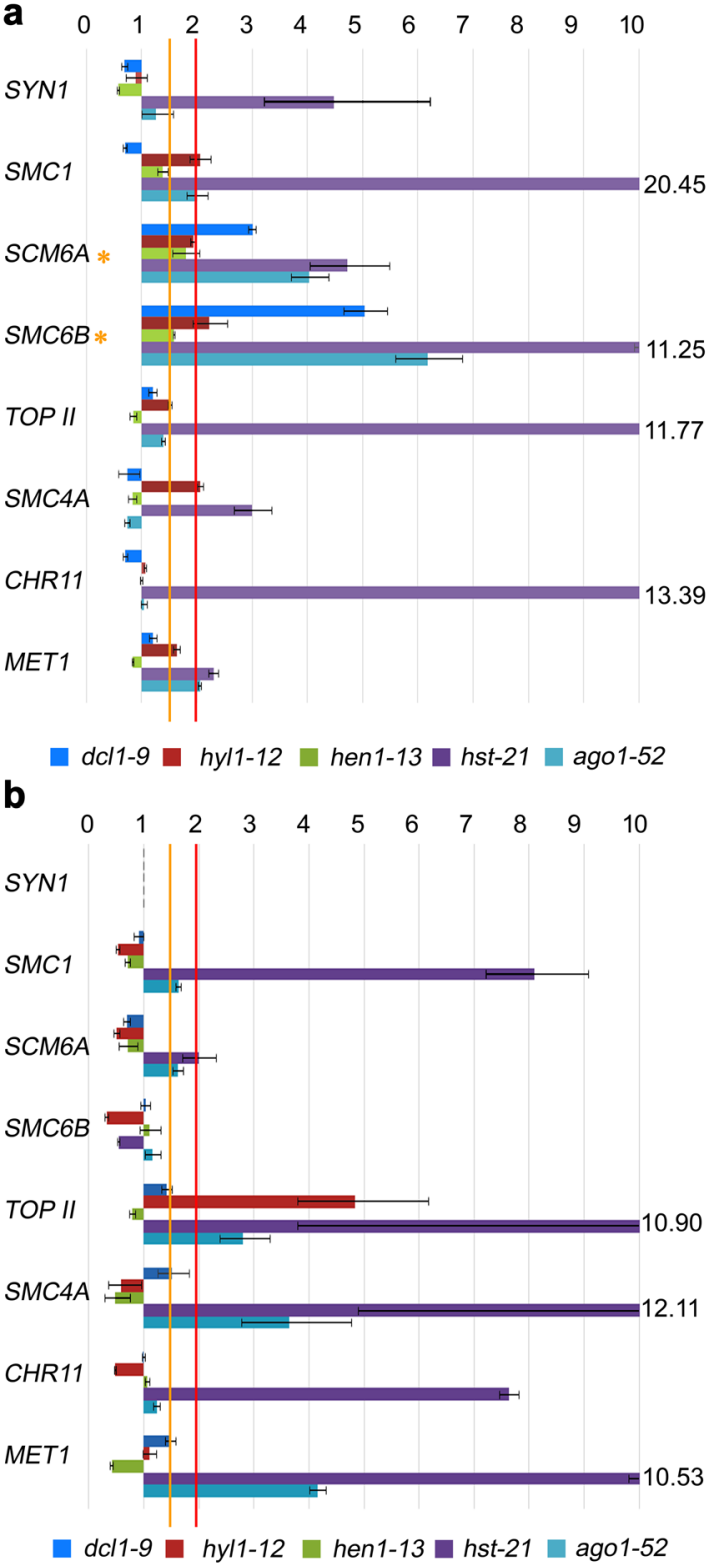
Relative expression assays of genes related to chromatin condensation. (**a**) Bud samples. (**b**) Leaf samples. Orange asterisks mark the genes that are overexpressed between 1.5- and 2.0-fold in all of the mutants compared with their wild types.

To ascertain the cause of the chromatin decondensation found in the mutants under study, we analyzed by quantitative RT-PCR (RT-qPCR) the expression of genes involved in chromatin structure maintenance or modification. We analyzed the expression of *SYNAPSIN 1* (*SYN1*), which encodes a meiotic-specific protein involved in sister chromatid cohesion and kinetochore orientation, and other genes with a role, both, at mitosis and meiosis: *STRUCTURAL MAINTENANCE OF CHROMOSOMES 1* (*SMC1*), an essential gene for sister chromatid cohesion; *SMC6A* and *SMC6B*, which encode components of the SMC5/6 complex involved in DNA damage response by HR; *TOPOISOMERASE II* (*TOPII*), with an essential function in crossover (CO) resolution in budding yeast, whose expression is increased in proliferative tissues; *SMC4A*, which forms part of the condensin complex; *CHROMATIN*-*REMODELING PROTEIN 11* (*CHR11*), implicated in cell proliferation during gametogenesis and sporophytic cell expansion; and *METHYLTRANSFERASE 1* (*MET1*), involved in CpG methylation. In order to obtain a general view about the expression profile of these genes in miRNA-machinery mutants, we isolated total RNA from two tissue types: buds (enriched in meiocytes) and leaves. Most of the genes analyzed displayed unexpectedly high mRNAs levels in both organs. *hst*-*21* and *ago1*-*52* had quite similar mRNA profiles and overexpressed all the genes analyzed in buds, and many of them in leaves (Fig. 4).

Several miRNAs have been detected in the male germline of Arabidopsis^8^. To investigate if the above-mentioned genes are miRNA targets, we used the psRNATarget Analysis Server (http://plantgrn.noble.org/psRNATarget/)^23^. Among 337 miRNAs in the psRNATarget database, miR5021 and miR161.2 were predicted to cleave the mRNA of *TOPII*, although the two target sites for miR5021 mapped at the 5′ UTR. In addition, miR866-5p was predicted to cleave the mRNA of *SMC4A*, and miR837-5p, to inhibit translation of this gene (Supplementary Table S3). miR5021 has been detected at very low level in pollen and considered a potential novel miRNA, because its genomic locus encodes an stem-loop miRNA precursor^8^. miR161.2 has been experimentally validated, targeting mRNAs of genes encoding proteins with pentatricopeptide repeats^24^. Degradome data support miR-837-5p as a true miRNA whose validated target is the mRNA of the gene encoding the Factor of DNA methylation 1 (FDM1), which is involved in RNA-directed DNA methylation^25^. miR866-5p co-immunoprecipitates at very low levels with AGO1 and has been identified in flower- and leaf-tissue small RNA libraries^26^.

### Chiasma frequency is increased in miRNA-machinery mutants

Meiotic COs, manifested cytologically as chiasmata, are essential for fertility because they ensure correct segregation of homologous chromosomes at anaphase I. We examined chiasma frequency in miRNA-machinery mutants. To determine the number of chiasmata corresponding to each chromosome, we performed fluorescence *in situ* hybridization (FISH) using probes against 45S and 5S rDNA repeats, which reside on chromosomes 2 and 4 (45S rDNA) and on chromosomes 3, 4 and 5 (5S rDNA)^27^. We found slight but statistically significant differences in several mutants (Table 1), which resulted from an increase in the number of chiasmata in the short arms of chromosomes 2 and 4, and in both arms of chromosome 5. Some mutants also showed a significant increase in chiasma frequency in the long arm of chromosome 2 (*dcl1*-*9*) and occasionally in the short arm of chromosome 3 (*hen1*-*6*; Table 1

**Table 1:** Mean chiasma frequencies per cell, per bivalent and per bivalent arm (short vs. long).

	Bivalents										C	n
	1		2		3		4		5			
	s	l	s	l	s	l	s	l	s	l		
L*er*	—	—	0.56	1.02	0.97	1.00	0.52	0.95	0.86	1.16	9.38	63
	2.35 (0.25)		1.57 (0.17)		1.97 (0.21)		1.48 (0.16)		2.02 (0.21)			
*dcl1-9*	—	—	0.71	1.17*	0.98	1.10	0.76**	1.05	1.00**	1.34*	10.59***	59
	2.47 (0.23)		1.88** (0.18)		2.08 (0.20)		1.81** (0.17)		2.34** (0.22)			
*hyl1-12*	—	—	0.74*	1.00	0.98	1.00	0.72*	1.02	0.94	1.20	10.02**	50
	2.42 (0.24)		1.74 (0.17)		1.98 (0.20)		1.74* (0.17)		2.14* (0.21)			
*hen1-13*	—	—	0.61	1.00	0.93	1.07	0.59	1.06	0.94	1.27	9.91*	70
	2.44 (0.25)		1.61 (0.16)		2.00 (0.20)		1.64 (0.17)		2.21* (0.22)			
*hst-21*	—	—	0.78*	1.04	0.98	1.02	0.78**	0.98	0.96	1.29	10.20***	45
	2.38 (0.23)		1.82* (0.18)		2.00 (0.20)		1.76** (0.17)		2.24** (0.22)			
*ago1-52*	—	—	0.52	1.12	0.88	1.07	0.69	1.03	0.81	1.21	9.78	67
	2.45 (0.25)		1.64 (0.17)		1.96 (0.20)		1.72** (0.18)		2.01 (0.21)			
Col-0	—	—	0.61	1.14	0.90	1.26	0.48	1.01	0.97	1.30	10.20	69
	2.52 (0.25)		1.75 (0.17)		2.16 (0.21)		1.49 (0.15)		2.28 (0.22)			
*hyl1-2*	—	—	0.75	1.10	0.96	1.21	0.65	1.13	1.00	1.46	10.75*	48
	2.50 (0.23)		1.85 (0.17)		2.17 (0.20)		1.77* (0.16)		2.46 (0.23)			
*hen1-6*	—	—	0.63	1.13	1.00**	1.50	0.63	1.00	1.00	1.25	10.88	8
	2.75 (0.25)		1.75 (0.16)		2.50 (0.29)		1.63 (0.15)		2.25 (0.21)			
*hst-17*	—	—	0.79*	1.13	0.90	1.25	0.58	1.08	0.94	1.33	10.60	48
	2.60 (0.25)		1.92 (0.18)		2.15 (0.20)		1.67 (0.16)		2.27 (0.21)			

C: mean cell chiasma frequencies. n: number of cells. s: short arm. l: long arm. For each genotype, the upper row indicates the mean chiasma frequency per bivalent arm (s and l); the bottom row includes the mean chiasma frequency per bivalent (s + l), and the contribution to the total chiasma frequency (as proportion of total cells) in parentheses. Asterisks indicate significant differences with the corresponding wild type in a Student’s *t*-test (*p < 0.05; **p < 0.01; ***p < 0.001).

).

### Genes involved in homologous recombination are up-regulated in miRNA-machinery mutants

**Figure 5:**
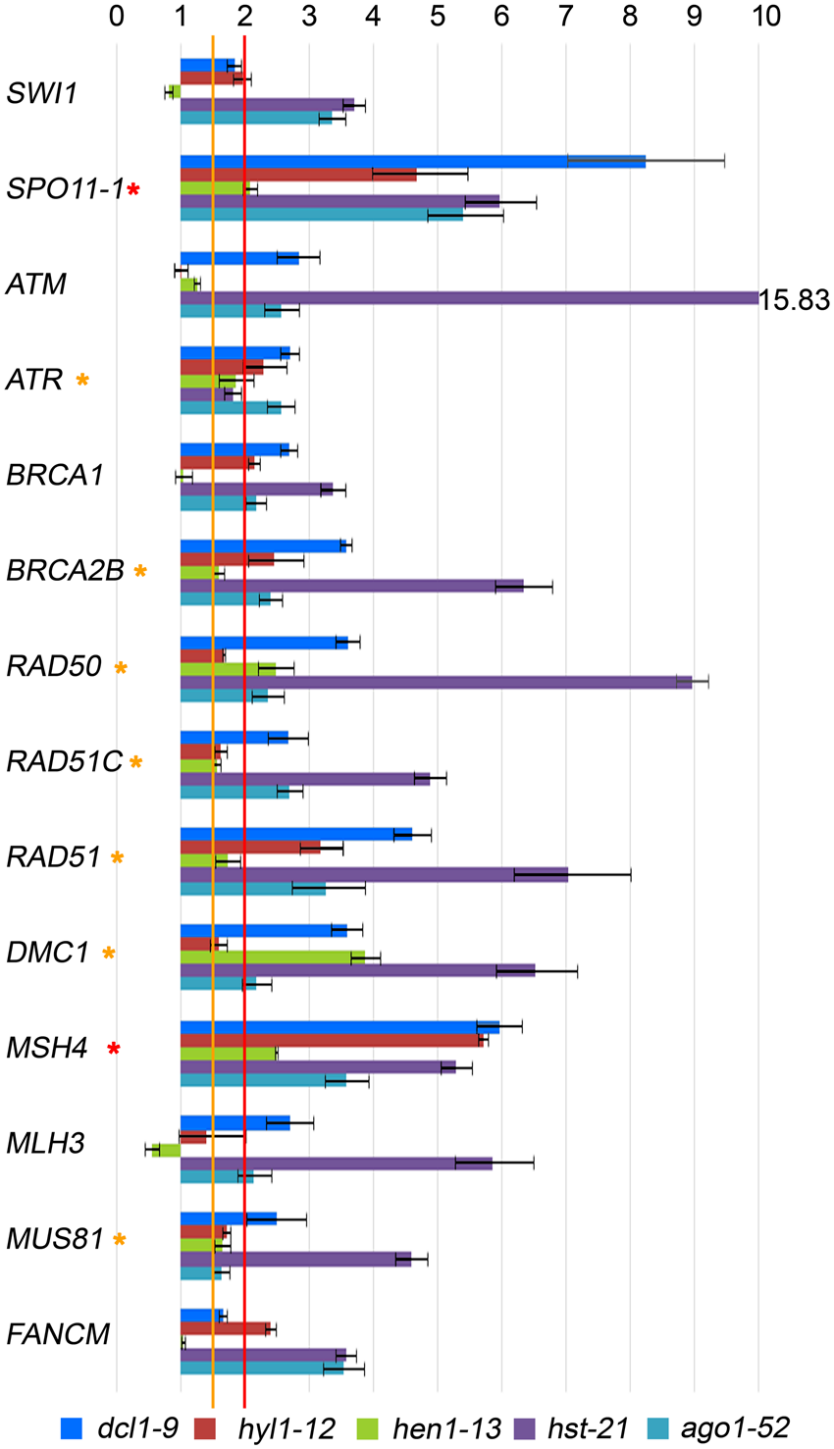
Relative expression assays of some genes related to homologous recombination in bud samples. Red asterisks mark the genes that are overexpressed more than two-fold in all of the mutants compared with the wild type. Orange asterisks mark the genes that are overexpressed 1.5- to 2.0-fold.

**Figure 6:**
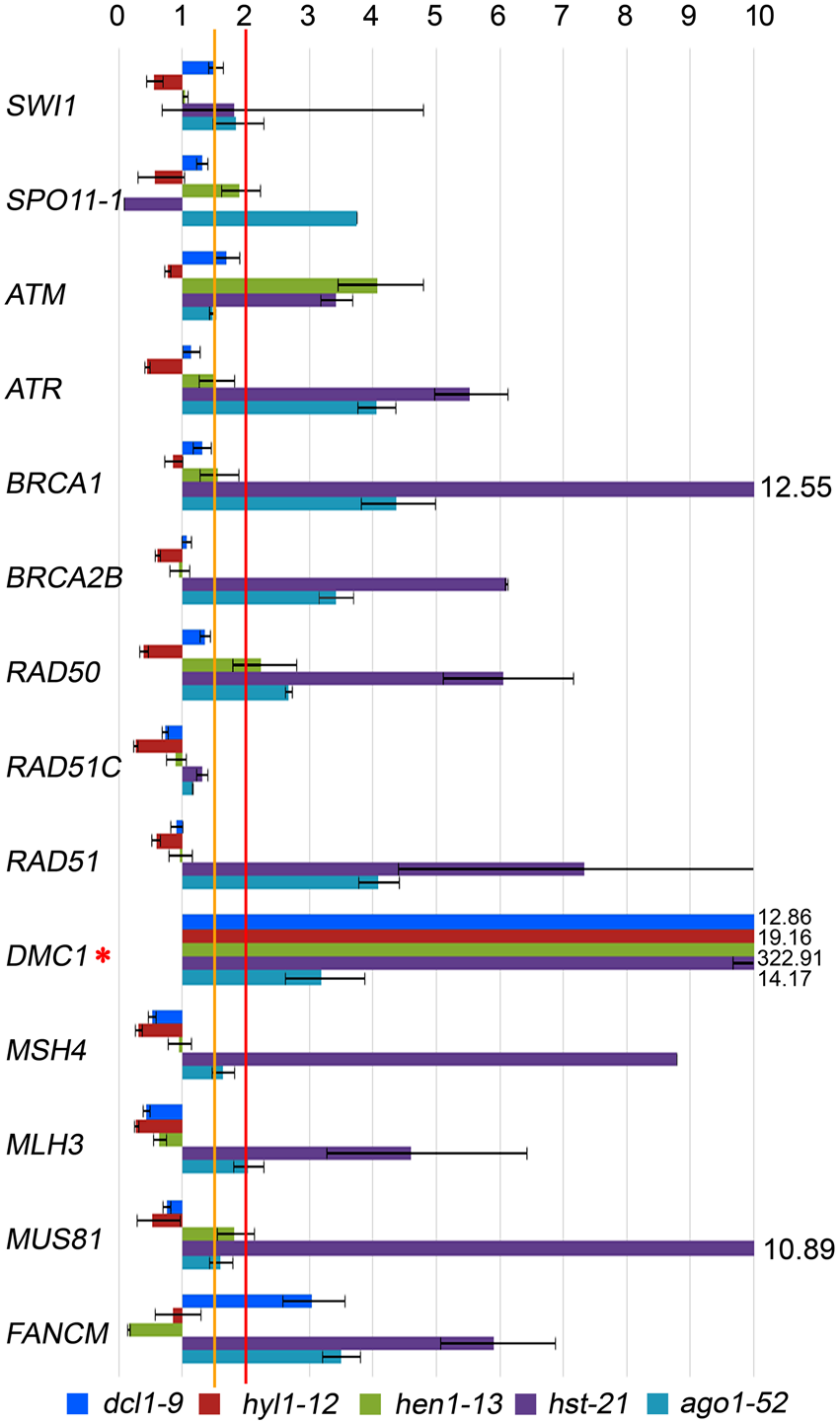
Relative expression assays of genes related to homologous recombination in leaf samples. The red asterisk marks the *DMC1* gene, which is overexpressed more than two-fold in all of the mutants compared with their wild types.

To determine whether the observed increase in chiasma frequency results from de-regulation of genes involved in HR, we studied the genes mentioned below. *SWITCH1* (*SWI1*) encodes a chromatin remodeling protein involved in the beginning of meiotic recombination and sister chromatid cohesion. *SPORULATION11*-*1* (*SPO11*-*1*) encodes a topoisomerase involved in the formation of programmed DNA double-strand break (DSB) during early meiosis. *ATAXIA*-*TELANGIECTASIA MUTATED* (*ATM*) and *ATAXIA TELANGIECTASIA*-*MUTATED AND RAD3*-*RELATED* (*ATR*) encode two kinases involved in the phosphorylation of H2AX (γH2AX) at DSB sites. *BREAST CANCER SUSCEPTIBILITY1* (*BRCA1*) and *BRCA2B* encode proteins involved in repairing damaged DNA. *RAD50* encodes a component of the MRN complex (MRE11–RAD50–NBS1), which processes the DSBs to produce single-stranded DNA (ssDNA) ends where recombinases bind. *RAD51C* participates in DSB repair by HR. *RAD51* encodes the main recombinase implicated in the repair of DSBs by HR, whereas *DMC1* is the meiosis-specific homologue of *RAD51*. *MutS HOMOLOG 4* (*MSH4*) and *MutL PROTEIN HOMOLOG 3* (*MLH3*) encode two components of the pathway that leads to CO formation subjected to positive interference. *MMS AND UV SENSITIVE 81* (*MUS81*) and *FANCONI ANEMIA COMPLEMENTATION GROUP M* (*FANCM*) encode antagonistic proteins acting in the pathway that leads to CO formation insensitive to interference. Once again, we examined RNA samples from buds and leaves, since HR is required for the repair of either DSBs that arise during the cell cycle or programmed DSB produced by SPO11 in meiocytes. We detected increased levels of mRNAs, compared to the wild-type plants, for most of these genes in buds and leaves, although with variations among different mutants. *hst*-*21* showed the highest differences, and again *ago1*-*52* and *hst*-*21* displayed similar expression patterns, mainly in reproductive tissues (Figs 5 and 6).

We also searched for miRNAs that might target the mRNAs of the above-mentioned genes, which are involved in HR and were found overexpressed in the mutants. According to psRNATarget, only two of those genes are predicted to be targeted by miRNAs, in both cases to cleave their mRNAs: *SWI1* by miR414 and miR426, and *MUS81* by miR854a-e (Supplementary Table S3). Mature miR854 is found in Arabidopsis rosette leaves, stems and flowers of wild-type plants, but not in those of *dcl1*, *hyl1* and *hen1* mutants^28^. It needs to be noted, however, that miR414 and miR426 are questioned as true miRNAs in the miRBase.

### *DMC1* is overexpressed in *hen1-13*

An unexpected result of our RT-qPCR analyses was the overexpression of the meiotic-specific recombinase *DMC1* in all the mutants studied, not only in buds, but also in leaves. The expression level was especially high in leaves of *hen1*-*13* (Fig. 6). Despite its meiotic-specific function, *DMC1* is overexpressed in somatic tissues of the *ddm1* mutant, which is defective for the DECREASE IN DNA METHYLATION 1 (DDM1) protein. *DMC1* is also induced after treatment with the DNA methylation inhibitor 5-aza-2′deoxicytidine (5-AC)^29^. DMC1 forms foci with RAD51 at DSBs during meiosis. To examine the effects of an excess of *DMC1* mRNA in meiosis, we performed a DMC1 immunolocalization in *hen1*-*13* plants, but no differences with L*er* were seen in the number of DMC1 foci (Supplementary Fig. S10). Therefore, either the excess mRNA produced or accumulated in the *hen1*-*13* mutant is not translated to protein or, at least, the DMC1 protein does not form extra foci in our experimental conditions.

To determine if *DMC1* overexpression could be associated to a defect in DNA repair, we tested the sensitivity of *hen1*-*13* to gamma irradiation, which is known to cause severe genome damage, mainly DSBs. *hen1*-*13* and L*er* seeds were able to germinate after being irradiated at four different doses, but the mutant was significantly more sensitive at 300 Gy. At this dose, the mutant seeds germinated and the seedlings expanded their cotyledons, but they failed to produce leaves. At a higher dose (450 Gy), both L*er* and *hen1*-*13* seedlings failed to produce leaves (Supplementary Fig. S11).

RAD51 is involved in the repair of somatic DSBs, generated by DNA damage, as well as meiotic-programmed DSBs. The meiotic phenotype of the *hen1*-*6 rad51*-*3* double mutant (both mutations are null and in a Col-0 background) was stronger than that of the *rad51*-*3* single mutant, with higher levels of chromosome fragmentation at metaphase I-anaphase I (Supplementary Fig. S12), notwithstanding *DMC1* is overexpressed in these plants. Furthermore, the double mutant showed trichomes with more than three branches, suggesting alterations in the endoreduplication control^30^. Increased endoreduplication should result from high levels of HR^31^. Altered trichomes were not observed in the single mutants (Supplementary Fig. S13).

## Discussion

### Loss-of-function alleles of miRNA-machinery genes cause semi-sterility in pollen mother cells

Many miRNA targets are mRNAs encoding transcription factors that control important developmental processes, such as organ patterning and development, flowering time, hormone regulation and meristem function^20^. Hence, mutations that damage miRNA-machinery components often lead to pleiotropic phenotypes^19, 32, 33^. In order to ascertain if the miRNA machinery participates in Arabidopsis meiosis and fertility, we studied these processes in *ago1*, *hyl1*, *hen1*, *dcl1* and *hst* mutants. We found that the reproductive morphological and cytological phenotypes correlate in some, but not all these mutants. The complete sterility of *dcl1*-*9* and *ago1*-*101* might be an indirect consequence of abnormal development of their reproductive organs (Fig. S2). Indeed, the developmental programs controlling the number and morphology of reproductive organs are perturbed in the *dcl1*-*9* mutant^19^ (Supplementary Figs S1 and S2). Similarly, the *ago1*-*101* mutant produces inflorescences with aberrant flowers, including anthers that lack PMCs (Supplementary Fig. S2). The reduced fertility observed in the remaining mutants studied here cannot be attributed to developmental defects of their reproductive organs, whose morphology is indistinguishable from wild type. All of the miRNA-machinery mutants studied showed a decrease in the number of PMC and MMC per bud, assuming that there is a single MMC per embryo-sac. This reduction, which was more pronounced for the PMCs than the MMCs (Fig. 1), does not seem to be a direct consequence of meiotic alterations: no alterations in pairing, synapsis and homologous chromosome segregation were seen (Supplementary Figs S3 and S4). PMCs and MMCs originate from archesporial cells^34^. Genome instability produced by chromatin alterations in archesporial cells could affect premeiotic cell divisions. Certainly, such genome instability might be more severe for the male germline, since multiple mitoses are needed for the production of PMCs, whereas the female archesporial cell directly functions as a MMC^35, 36^.

Although the AGO1, DCL1, HST and HEN1 proteins are also involved in small RNA pathways other than that of miRNAs^24^, our results suggest that some miRNAs regulate germ-cell and somatic specification in anthers. Supporting this notion, loss-of-function alleles of genes involved in germ-cell line specification, such as *BARELY ANY MERISTEM 1* and *2* (*BAM1* and *BAM2*), *EXCESS MICROSPOROCYTES 1* (*EMS1*), and *SOMATIC EMBRYOGENESIS RECEPTOR*-*LIKE KINASE 1* and *2* (*SERK1* and *SERK2*), display increased PMC numbers^37^. In addition, bioinformatic analyses suggest that *BAM2*, *EMS1* and *SERK2* might be regulated by miRNAs^38^. Transcripts from *ARF6* and *ARF8*, encoding two Auxin Response Factors essential for correct gynoecium and stamen development, are targeted by *miR167* ^39^. Some other genes that control floral development, such as *UNUSUAL FLORAL ORGANS* (*UFO*), *BLADE ON PETIOLE 1* (*BOP1*), *APETALA 2* (*AP2*) and *TERMINAL FLOWER 2* (*TFL2*)^40–42^, are predicted to be miRNA targets. Changes in the expression of one or more of these genes might explain the decrease in PMCs exhibited by the mutants studied here.

### Loss-of-function mutations of miRNA-machinery genes affect chromatin condensation during first meiotic division

Histone modifications generate different chromatin configurations involved in a variety of basic cellular functions, such as transcriptional activity, DNA replication and repair, and chromosome recombination and segregation^21^. Previous studies have revealed the presence of chromatin condensation defects in mutants defective for RNA-directed DNA methylation (RdDM) pathway, in somatic cells during interphase^43^ and in post-meiotic male gametophytes^44^. Recently, we have also demonstrated that chromatin is disturbed in PMCs from mutant plants defective for several proteins related to RdDM. Here, our cytological observations suggest that genes of the miRNA machinery are important for maintaining chromatin structure and chromosome organization during early meiotic stages (Fig. 2, Supplementary Table S2). Indeed, different types of small non-coding RNAs have been shown to participate in DNA and histone methylation^45^. Epigenetic silencing of transposable elements (TEs) is mediated by RNA-dependent DNA methylation (RdDM) guided by siRNAs. When TEs are epigenetically reactivated during germline reprogramming, TE transcripts are targeted by epigenetically activated small interfering RNAs (easiRNAs), whose biogenesis is directed by miRNAs^46, 47^. In fact, thousands of TEs were found up-regulated in isolated meiocytes in two RNA-seq searches for meiosis-specific genes^48, 49^, which highlights the importance of small RNA and miRNA-mediated regulation of meiosis in flowering plants. In addition, trans-acting small interfering RNAs (ta-siRNAs), which are generated after miRNA-directed cleavage of *TAS* gene transcripts, guide DNA methylation of *TAS* loci by RdDM. *DCL1* is one of the genes involved in ta-siRNA-mediated RdDM^50^.

We found increased levels of mRNAs from genes involved in chromatin structure maintenance, which could be due to the direct or indirect action of miRNAs on these mRNAs. A recent analysis of 338 mature Arabidopsis miRNAs from the Plant microRNA Database (PMRD; http://bioinformatics.cau.edu.cn/PMRD)^38^ identified 2,862 potential targets^51^. These targets include several genes encoding proteins involved in histone modification, DNA methylation and chromatin remodeling, including *SMC6A*, *TOPII* and *SWI1*, whose mRNAs we found accumulated in the mutants studied here. In addition to *SMC6A*, other predicted target genes belong to the SMC family: *SMC1* and *SMC3*, which encode proteins of the cohesin complex; and *SMC2 a*nd *SMC4*, which encode proteins of the condensin complex^52^. We found high levels of mRNAs from these *SMC* genes in bud samples of some of the mutants analyzed (Fig. 4). For example, the level of *SMC1* mRNA detected in *hst*-*21* was about 24-fold higher than that of L*er*, and all mutants showed an increase of *SMC6A* and *SMC6B* mRNA amounts. SMC6A and SMC6B, together with SMC5, participate in DSB repair by HR^53^. Some other predicted miRNA targets encode proteins with a SWI domain, which are ATP-dependent and modify chromatin structure, modulating the access of transcription factors to DNA. For instance, the *SWI1* gene encodes a protein involved in chromatin structure maintenance, sister chromatid cohesion establishment and axial element formation during meiosis^54^. Taken together, these prior observations and the results presented here suggest that the de-regulation of genes involved in chromatin structure maintenance, whose RNAs accumulate in the miRNA-machinery mutants studied here, cause the chromatin decondensation shown by these mutants.

### Key genes of homologous recombination are up-regulated in miRNA-machinery mutants

High mRNA levels of the meiotic-specific genes *SPO11*-*1* and *MSH4* were found in buds of the mutants that we analyzed (Fig. 5). A more open chromatin conformation during prophase meiotic stages together with a high concentration of the SPO11-1 protein could cause an increase in the number of DSBs^55^. A subsequent overexpression of genes encoding proteins involved in the ulterior steps of the HR process would lead to a slight increase of reciprocal recombination events. This hypothesis might explain the increased mean cell chiasma frequency observed in *dcl1*-*9*, *hyl1*-*2*, *hyl1*-*12*, *hen1*-*13* and *hst*-*21* (Table 1). A variation in chiasma frequency has also been observed in the Arabidopsis *arp6* mutant, defective for a component of the chromatin remodeling complex SWR1-C^56^, in which the expression of several *MIR* genes is altered^57^. In this context, specific *cis*-regulatory elements have been characterized in several genes preferentially expressed during male meiosis^58^. Regarding the results obtained in other species, a novel miRNA isolated in maize meiocytes has a predicted target sequence in *RAD51C* ^9^ and *miR398* is more abundant in sunflower meiocytes from wild genotypes, which differ in recombination rates from domesticated genotypes^59^.

Accumulation of mRNAs from HR genes was also observed in leaf samples (Fig. 6). It is worth to mention that the number of miRNA targets might be underestimated in Arabidopsis. Between 30% and 60% of human genes are considered to be regulated by miRNAs, and some single miRNAs regulate more than one hundred genes^60^. Recent studies carried out in mammalian cell cultures have revealed that miRNA biogenesis is globally induced upon DNA damage in an ATM-dependent manner^61^. On the other hand, ATM and ATR are involved in the recruitment of the SMC5/SMC6 complex by H2AX phosphorylation, which might explain the increased *SMC6A* and *SMC6B* expression that we observed in miRNA-machinery mutants (Fig. 4). Taken together, our results show that components of the miRNA machinery are involved in the regulation of genes with effects on HR. Further studies will be required to determine whether these genes are direct or indirect targets of miRNAs.

The high overexpression of *DMC1* in the leaves of all the mutants analyzed, especially *hen1*-*6* (Fig. 6), was unexpected because *DMC1*, which encodes a meiosis-specific recombinase, has not been described as a miRNA target gene and its mRNA has not been previously found in leaf tissues. In fact, the *DMC1* promoter is regularly used to drive meiosis-specific expression^62, 63^. However, we detected *DMC1* expression in wild-type leaves (Fig. 6), and previous studies have suggested that the expression of this gene is not restricted to the germline^29, 64^. On the other hand, the excess of *DMC1* mRNA produced in *hen1*-*6* does not seem to be translated to protein (Supplementary Fig. S10).

Previous reports have demonstrated a link between the siRNA machinery genes *AGO2* and *AGO9* and DSB repair^15, 65^. In this study, we suggest the implication of *HEN1* in this process. This is supported by the *hen1*-*13* hypersensitivity to gamma-rays (Supplementary Fig. S11), and by the phenotype of the double mutant *hen1*-*6 rad51*-*3*, which displays increased endoreduplication in trichomes (Supplementary Fig. S13) and a higher level of chromosome fragmentation at metaphase I-anaphase I than that showed by *rad51*-*3* meiocytes (Supplementary Fig. S12).

In conclusion, we have found a link between loss- and lack-of-function mutations in genes of the miRNA pathway and chromatin decondensation during meiosis; these mutations were also associated to a general overexpression of genes involved in meiotic recombination and chromatin organization. Despite these defects, meiosis seems to proceed normally. Given that not few miRNA targets encode transcription factors, it is tempting to propose that the miRNA machinery regulates at least some of the transcription factors that bind to the promoters of the genes that control either meiosis entry or chromatin remodeling and dynamics during this division. This study extends previous knowledge, but further experiments will be required to identify these regulatory elements and to discover the functional mechanism by which the miRNA machinery influences on meiosis and fertility.

## Methods

### Plant materials and growth

Plants of the *Arabidopsis thaliana* (L.) Landsberg *erecta* (L*er*) and Columbia-0 (Col-0) accessions were obtained from NASC (Nottingham Arabidopsis Stock Centre). The *ago1*-*52* and *hst*-*21* mutations were induced by ethylmethane sulphonate (EMS), and *hen1*-*13* and *hyl1*-*12* by fast-neutrons, in a L*er* background^18, 66, 67^. NASC also provided the *dcl1*-*9* mutant, whose original background was Wassilewskija and was crossed five times to L*er* ^19^. T-DNA insertional mutants from the Salk Institute Genomic Analysis Laboratory (SiGnAL, http://signal.salk.edu/cgi-bin/tdnaexpress) were also provided by NASC: *ago1*-*101*, *dcl1*-*16*, *hen1*-*6*, *hst*-*17*, and *hyl1*-*2* (Supplementary Table S1). The latter mutants were genotyped by PCR using a T-DNA-specific primer, either LBb1.3 or LB2, and two gene-specific primers (Supplementary Table S4). All plants were grown on a mixture of vermiculite and commercial soil (3:1) and kept in a greenhouse under a 16-hour light/8-hour dark photoperiod, at 20 °C and 70% relative humidity.

### Fertility evaluation

To evaluate the extent of fertility of the mutants, we determined the number of seeds per silique, and the number of PMCs and MMCs. The number of seeds per silique was scored in three siliques from five plants of each genotype. The number of PMCs was determined in five buds from three plants of each genotype, which in all cases contained six anthers. The quantification of MMCs was carried out scoring embryonic sacs from squash preparations of five gynoecia from three plants of each genotype.

### Cytology

Fixation of flower buds, slide preparation of PMCs, and fluorescence *in situ* hybridization (FISH) were carried out as previously described^27^. Three plants of each mutant and their corresponding wild types were analyzed. Squash preparations were made removing gynoecia from fixed flowers. The gynoecia were transferred to a slide, stained with acetic carmine and squashed. Immunolocalization of modified histone H3 was carried out as previously described, with some modifications^22^. Immunolocalizations of ASY1 (1:1000), ZYP1 (1:500) and DMC1 (1:250) proteins were carried out as previously described^68^ with the primary antibodies shown in Supplementary Table S5. The secondary antibodies were anti-rabbit IgG FITC conjugated (1:50, Sigma) and anti-rat IgG Cy3 conjugated (1:50, Sigma).

### RT-qPCR

Total RNA was extracted with the RNeasy kit (Qiagen) from 100 mg of leaves and young buds of L*er*, *dcl1*-*9*, *hyl1*-*12*, *hen1*-*13*, *hst*-*21*, and *ago1*-*52*. qPCR was performed with the Transcriptor First Strand cDNA Synthesis kit and the FastStart TaqMan Probe Master kit using UPL (Roche, Universal Probe Library) probes, designed by the UPL Assay Design Center (https://lifescience.roche.com; Supplementary Table S6). The relative quantification of gene expression was monitored after normalization by the 18S rRNA as an internal control (Hs99999901_s1; Applied Biosystems, http://www.appliedbiosystems.com), considering fold variation over a calibrator (L*er*) using the ΔΔ*C*_T_ method.

### DNA damage sensitivity assays

Seeds from L*er* and *hen1*-*13* were kept in sterile water at 4 °C for 24 h and then exposed to 150, 300, and 450 Gy (with dose rates of 2.94 Gy/min) from a ^137^Cs source (IBL 437 C CIS bio International). Production of true leaves and fresh weight were scored 14 days after treatment.

### miRNA target prediction

The psRNATarget website (http://plantgrn.noble.org/psRNATarget)^23^ was used to check whether the mRNAs of selected genes (including *SWI1*, *DYAD*, *SPO11*, *ATM*, *ATR*, *BRCA1*, *BRCA2b*, *RAD50*, *RAD51*, *RAD51C*, *DMC1*, *MSH4*, *MLH3*, *MUS81*, *FANCM*, *SYN1*, *SMC1*, *SMC6A*, *SMC4*, *TOPII*, *CHR11* and *MET1*) are miRNA targets. Default settings were used, as previously described^69^. For target search, the “Arabidopsis thaliana, transcript, removed miRNA gene, TAIR, version 10, released on 2010_12_14” library was selected.

## Electronic supplementary material

Supplementary Material
