# Supplementary material for: Loss of function of Arabidopsis microRNA-machinery genes impairs fertility, and has effects on homologous recombination and meiotic chromatin dynamics
Source: Sci Rep. 2017 Aug 24;7:9280. doi: 10.1038/s41598-017-07702-x (PMC5571030; doi:10.1038/s41598-017-07702-x)
Supplement: Supplementary file 1 — Supplementary Material [file 41598_2017_7702_MOESM1_ESM.pdf]

Loss of function of Arabidopsis microRNA-machinery genes impairs fertility, and has effects on homologous recombination and meiotic chromatin dynamics

Cecilia Oliver, Mónica Pradillo, Sara Jover-Gil, Nieves Cuñado,
María Rosa Ponce and Juan Luis Santos

Supplementary Information

a

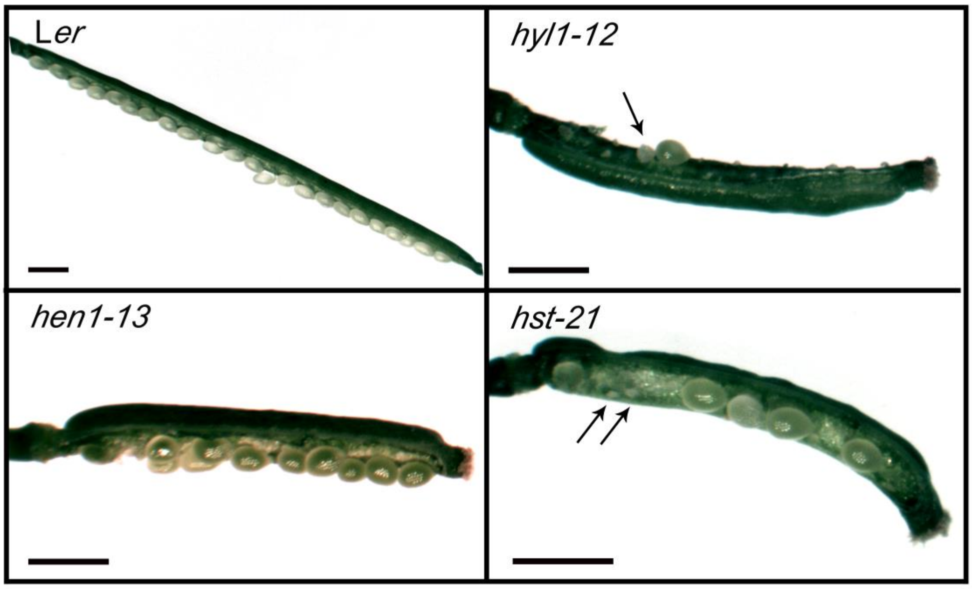

b

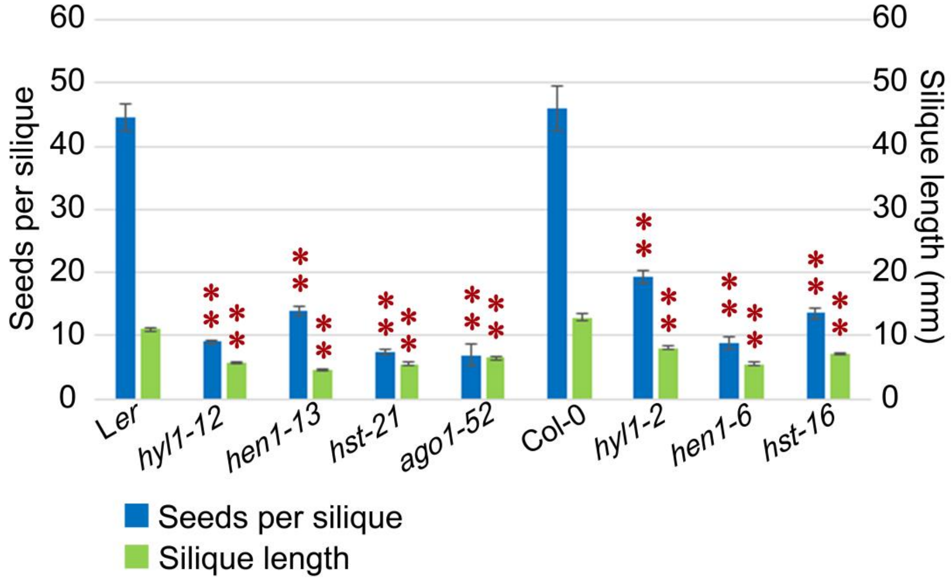

Supplementary Figure S1. Fertility in the *hyl1*, *hen1*, *hst* and *ago1* mutants. (**a**) Siliques from L*er, hyl1-12, hen1-13* and *hst-21.* Arrows point to aborted seeds. Scale bars: 1 mm. (**b**) Seeds per silique and silique length. Error bars indicate standard error of the mean. Asterisks indicate a significant difference with the corresponding WT in a Mann-Whitney U test (**p < 0.01).

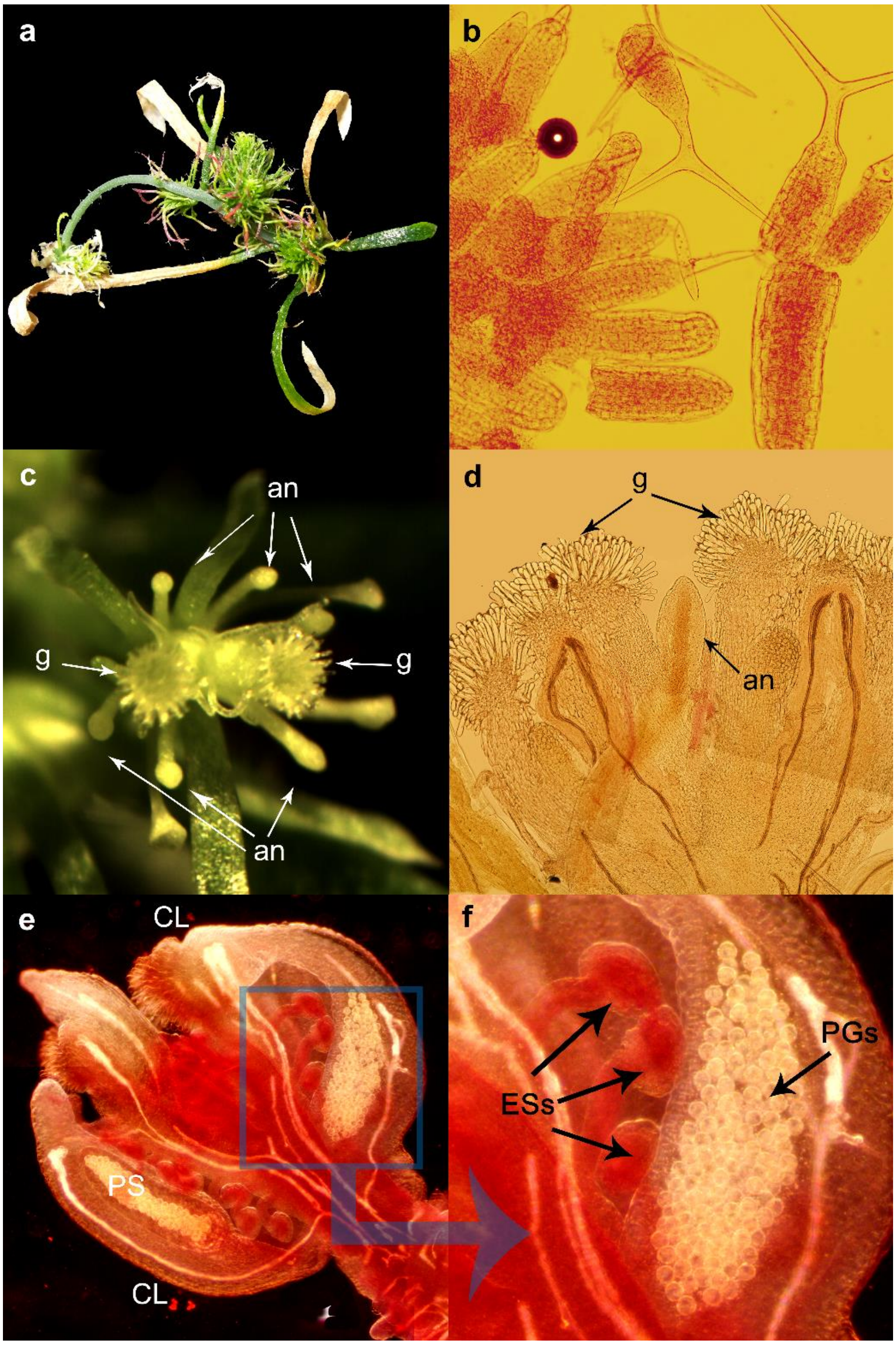

Supplementary Figure S2. Flower organ aberrations in *ago1-101* and *dcl1*-9. (**a**) *ago1-101* inflorescences formed by filamentous structures. (**b**) Detail of one of the filamentous structures shown in (a), stained with acetic carmine. (**c**) *ago1-101* flower displaying two gynoecia (g) and six anthers (an). (**d**) Acetic carmine staining of the flower shown in (b). (**e**) Acetic carmine staining of a *dcl1-9* gynoecium. (**f**) Magnification of (e), showing details of a pollen sac (PS) with pollen grains (PGs) within the carpel leaf (CL) and adjacent to the embryo sacs (ESs).

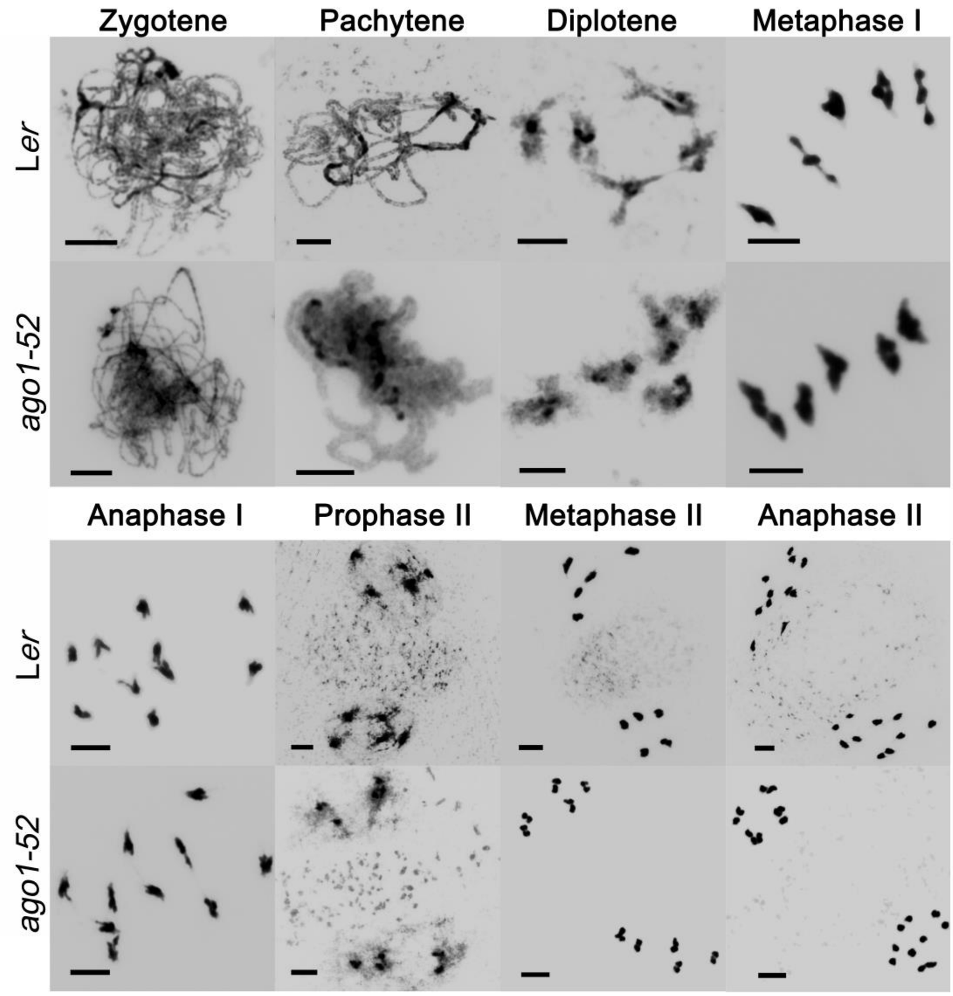

Supplementary Figure S3. Meiotic stages in PMCs from L*er* and *ago1-52.* Bars = 5 µm.

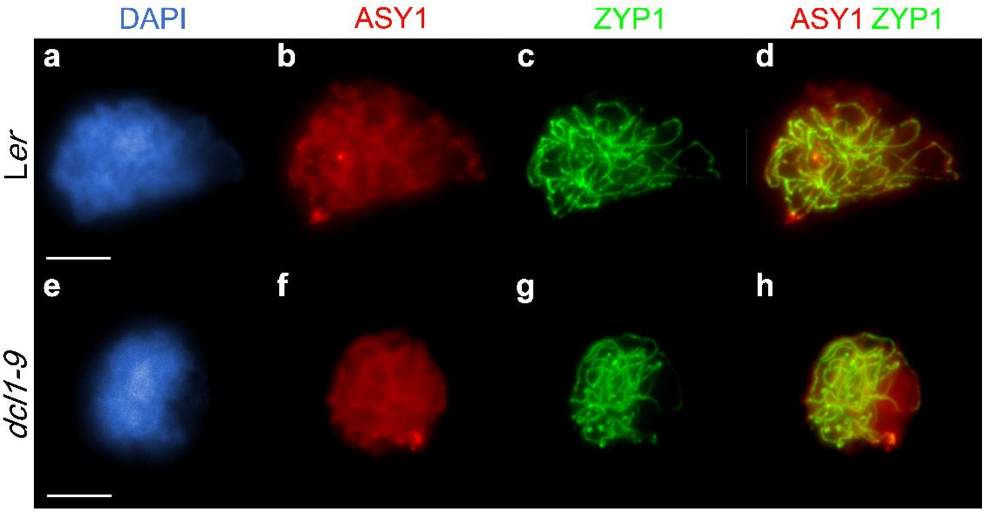

Supplementary Figure S4. ASY1 and ZYP1 immunolocalization in L*er* and *dcl1-9* meiocytes. (**a-d**) L*er* late pachytene. (**e-h**) *dcl1-9* late pachytene. Similar results were obtained with other mutants studied. Blue, red and green signals correspond to DAPI, ASY1 and ZYP1, respectively. Bars = 5 µm.

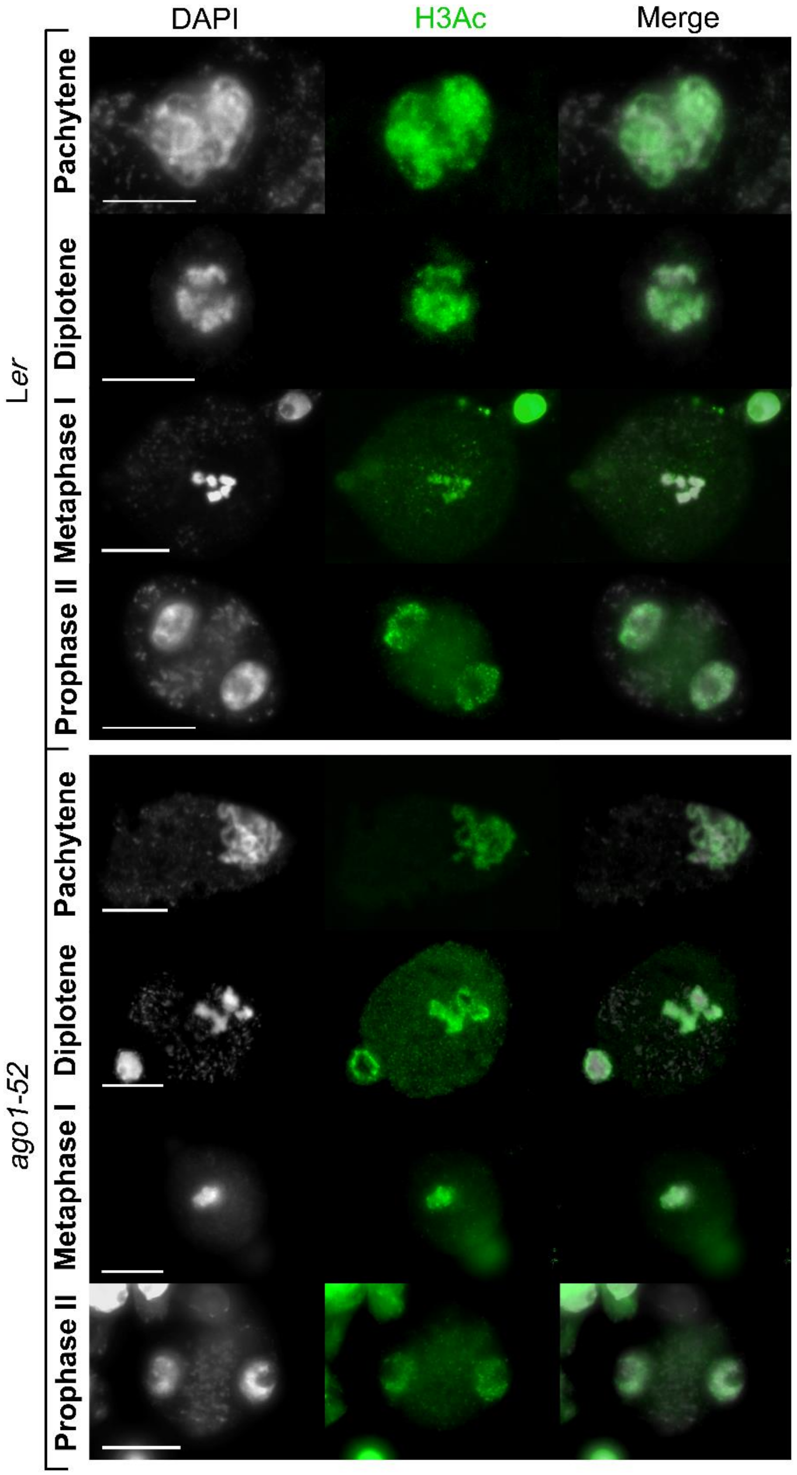

Supplementary Figure S5. Distribution pattern of H3Ac in PMCs from L*er* and *ago1-52.* Representative images of H3Ac immunolocalization, which marks euchromatin regions. Similar results were obtained with other mutants studied. Bars = 5 µm.

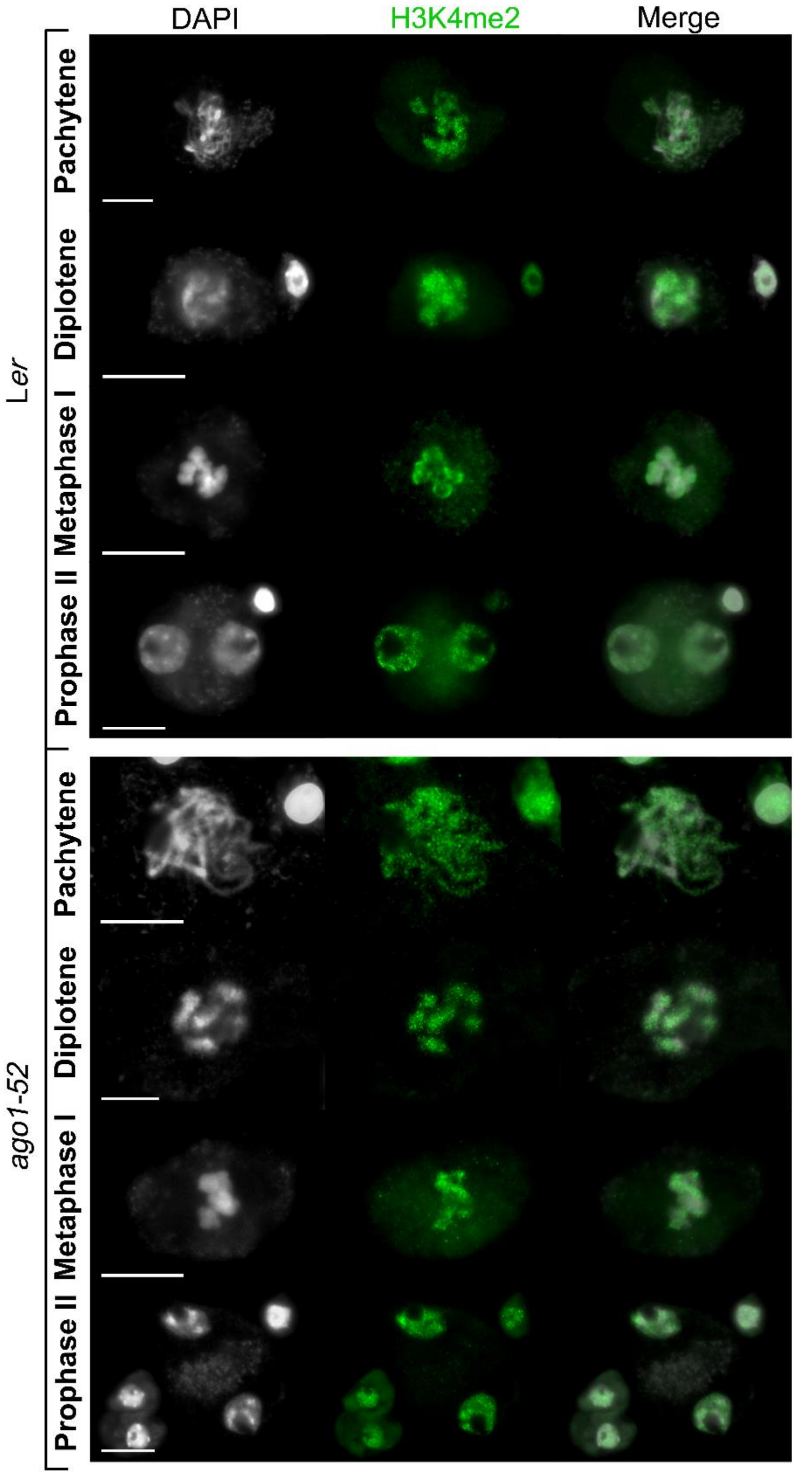

Supplementary Figure S6. Distribution pattern of H3K4me2 in PMCs from L*er* and *ago1-52*. Representative images of H3K4me2 immunolocalization, which marks euchromatin regions. Similar results were obtained with other mutants studied. Bars = 5 µm.

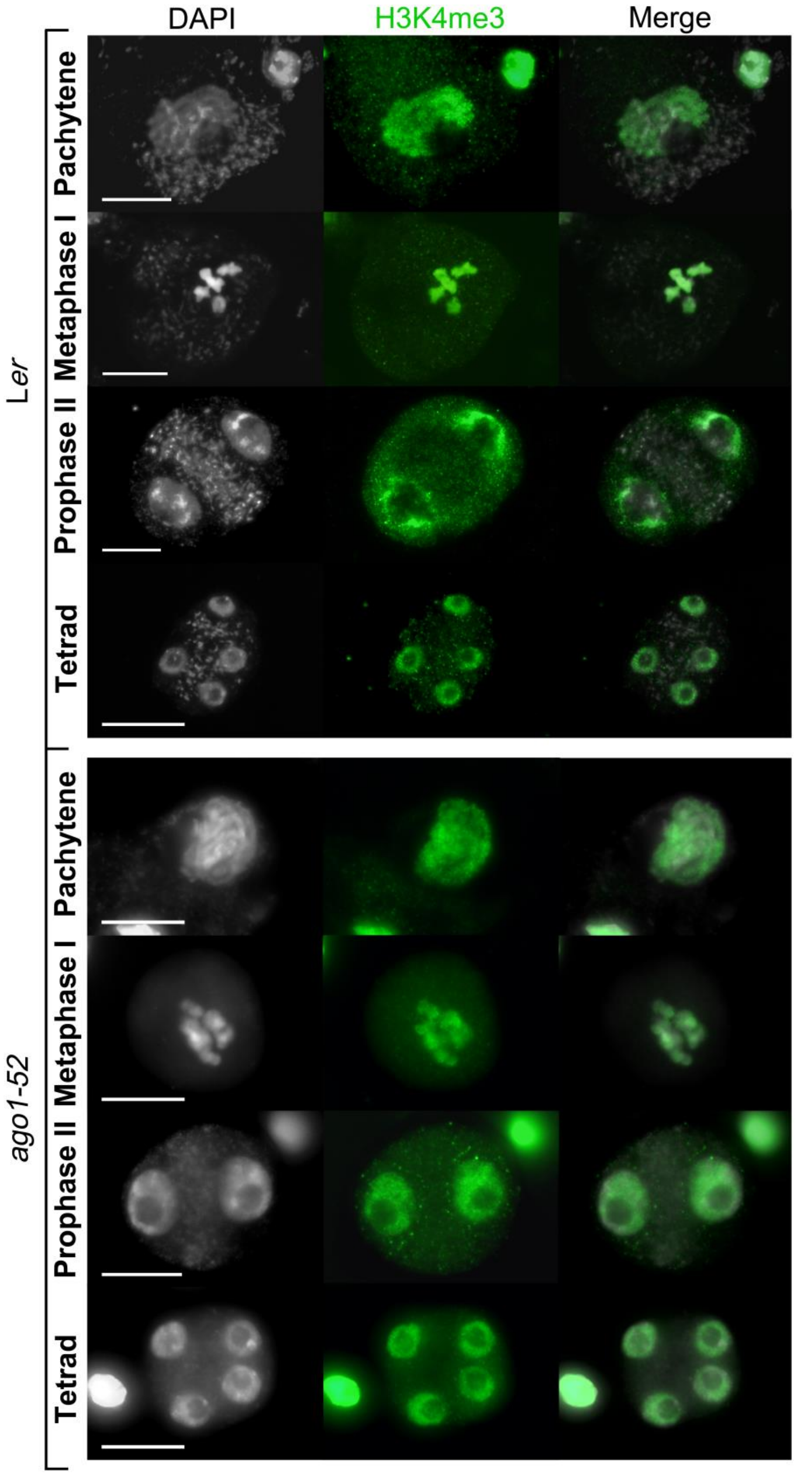

Supplementary Figure S7. Distribution pattern of H3K4me3 in PMCs from L*er* and *ago1-52.* Representative images of H3K4me3 immunolocalization, which marks euchromatin regions. Similar results were obtained with other mutants studied. Bars = 5 µm.

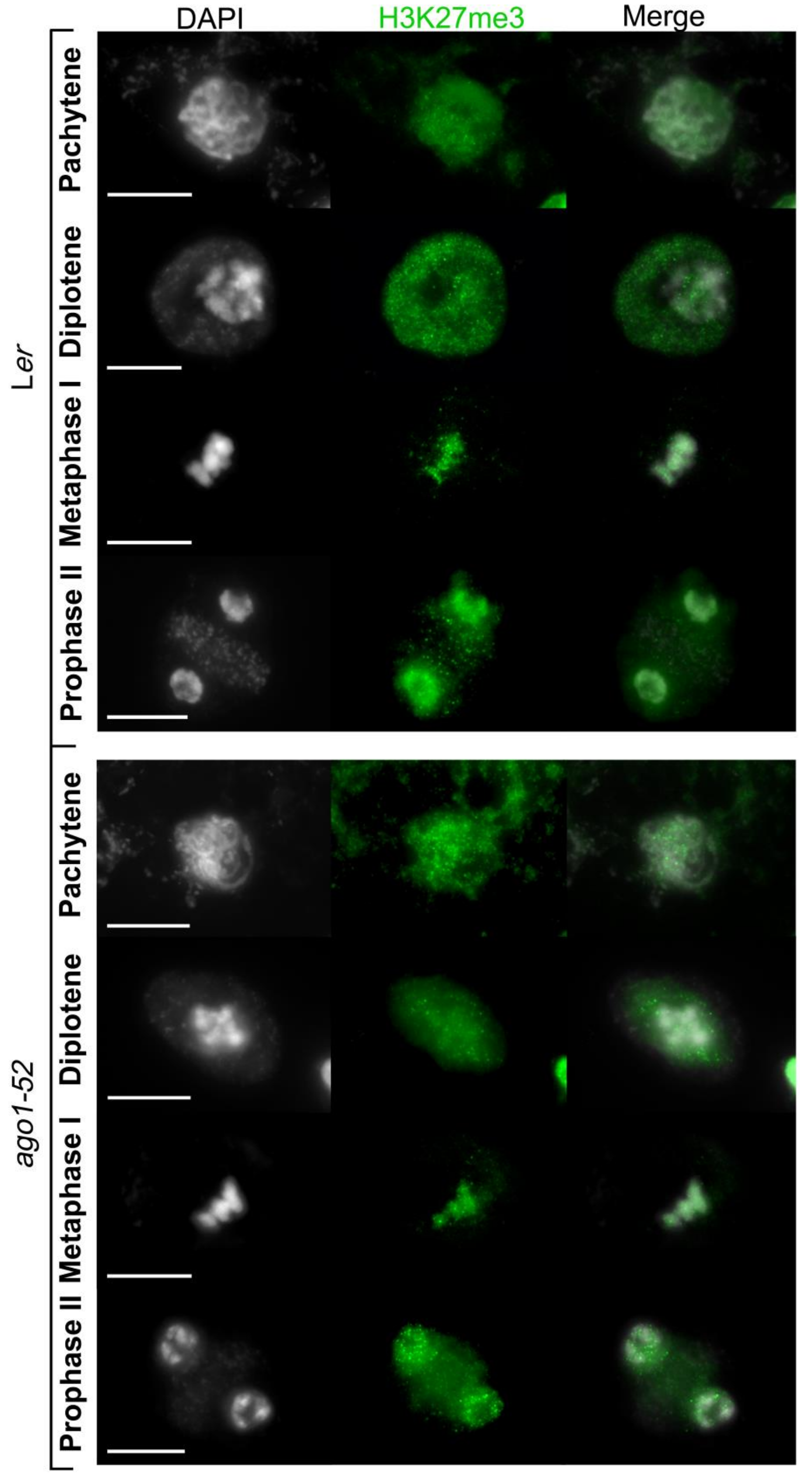

Supplementary Figure S8. Distribution pattern of H3K27me3 in PMCs from L*er* and *ago1-52.* Representative images of H3K27me3 immunolocalization, which marks euchromatin regions. Similar results were obtained with other mutants studied. Bars = 5 µm.

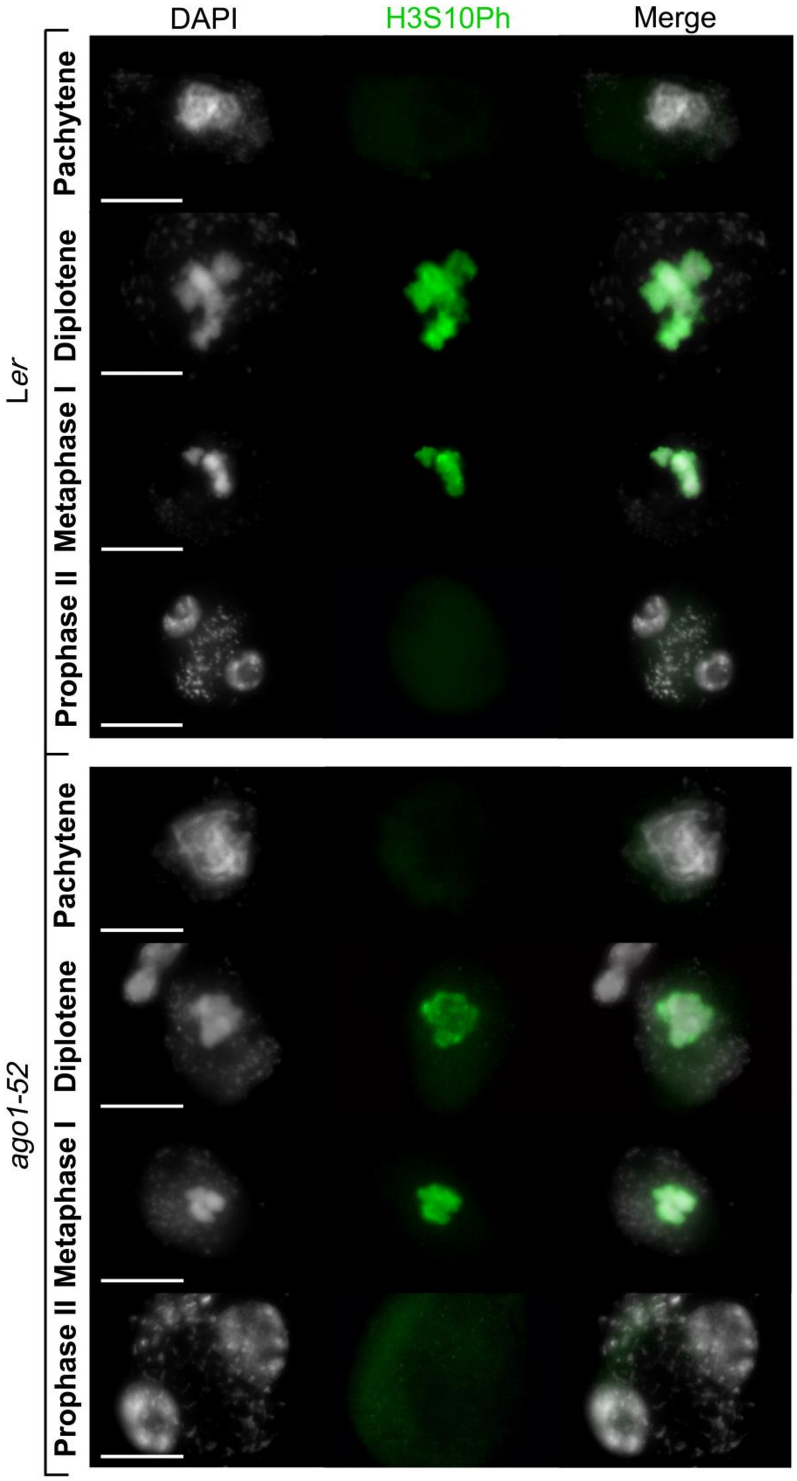

Supplementary Figure S9. Distribution pattern of H3S10Ph in PMCs from L*er* and *ago1-52.* Representative images of H3S10Ph immunolocalization, which marks condensed chromosomes. Similar results were obtained with other mutants studied. Bars = 5 µm.

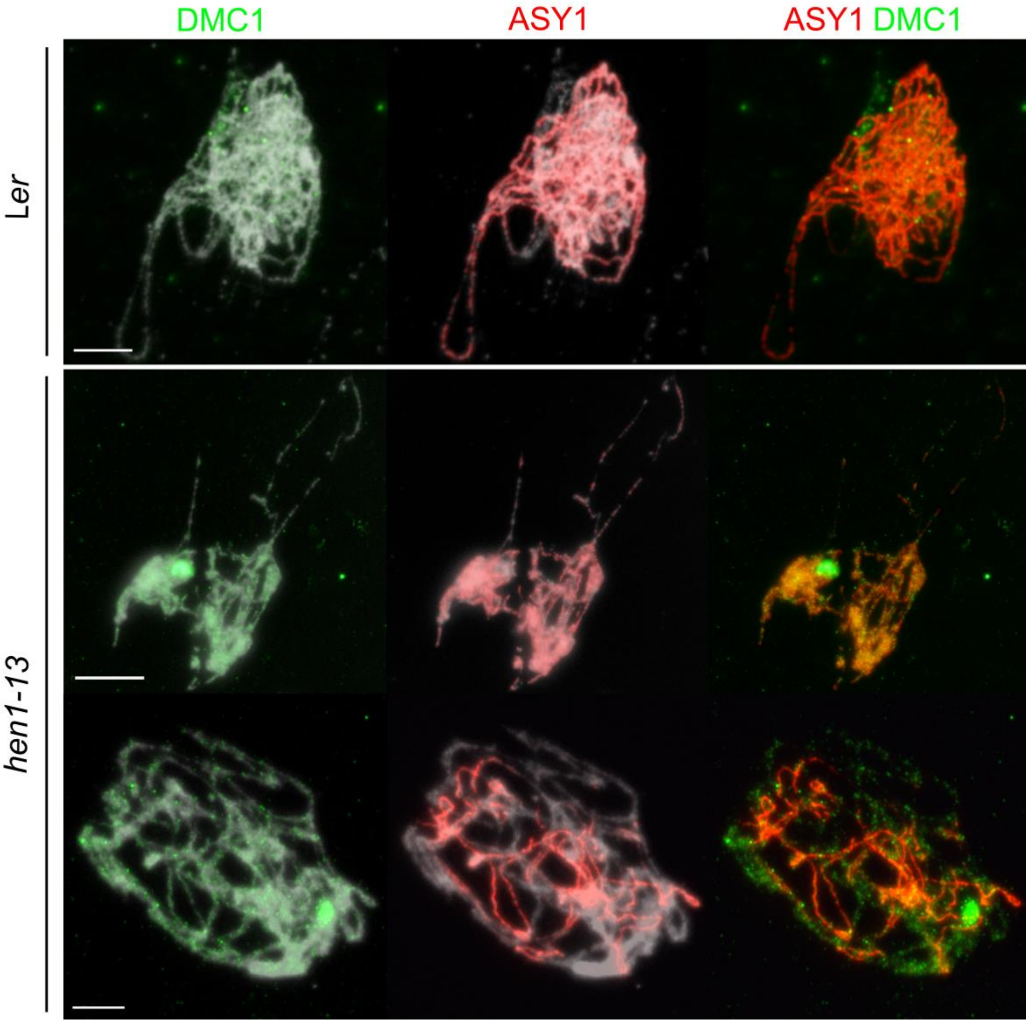

Supplementary Figure S10. DMC1 and ASY1 immunolocalization in L*er* and *hen1-13* meiocytes. (**a**-**c**) L*er*. (**d**-**i**) *hen1-13.* Green and red signals correspond to DMC1 and ASY1, respectively. Bars = 5 µm.

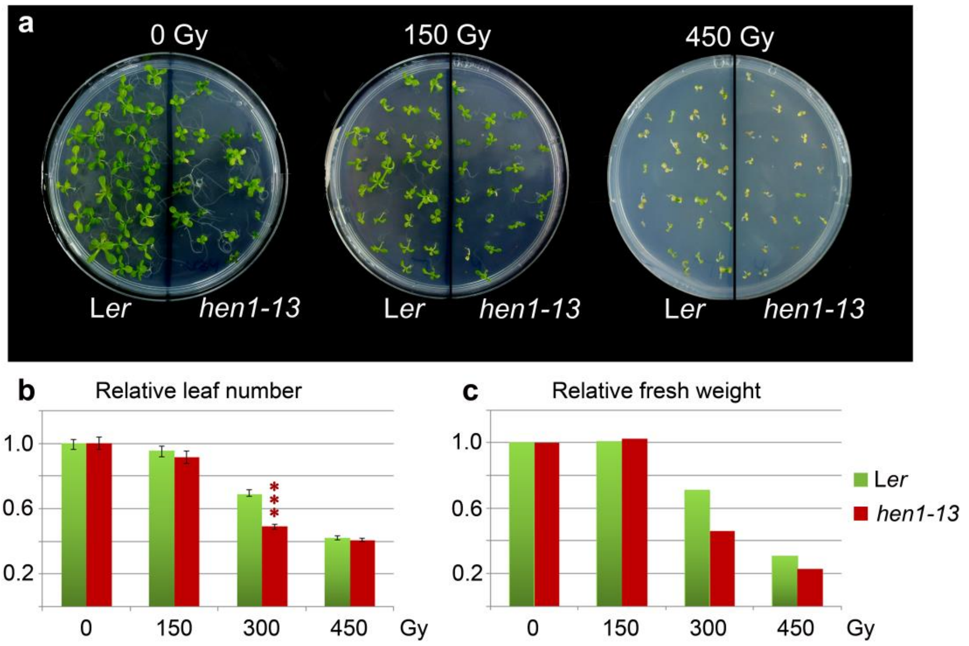

Supplementary Figure S11. Sensitivity to gamma rays in L*er* and *hen1-13*. (**a**) Representative images of plants exposed to 0, 150 and 450 Gy. (**b**) Relative leaf number after different doses of gamma irradiation. Leaf number was quantified in both genotypes and calculated in relation to the untreated control plants. Error bars indicate standard error of the mean. Asterisks indicate values significantly different from the corresponding wild type in a Mann-Whitney U test (***$p < 10^{-3}$). (**c**) Relative fresh weight after different doses of gamma irradiation. Fresh weight was measured in both genotypes and calculated in relation to the untreated control plants.

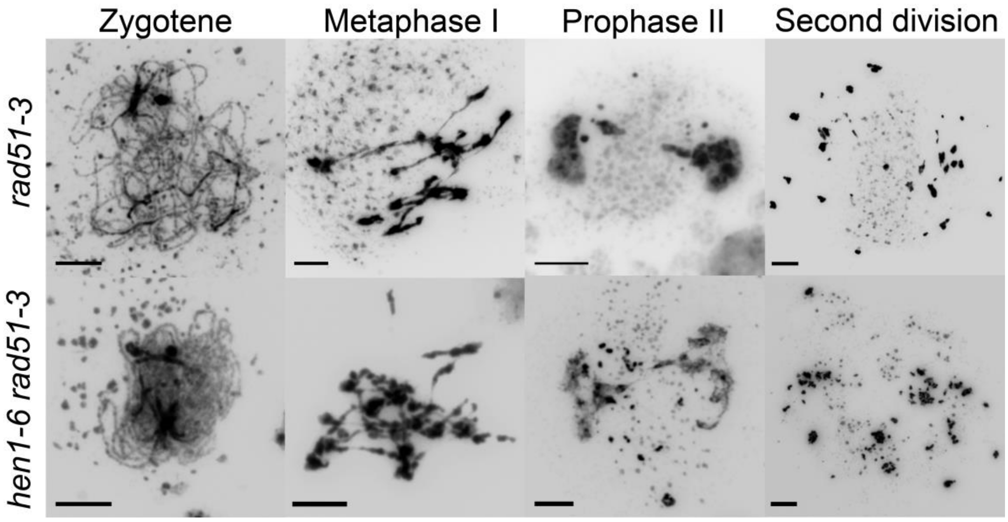

Supplementary Figure S12. PMCs from *rad51-3* and *hen1-6 rad51-3.* Bars = 5 µm.

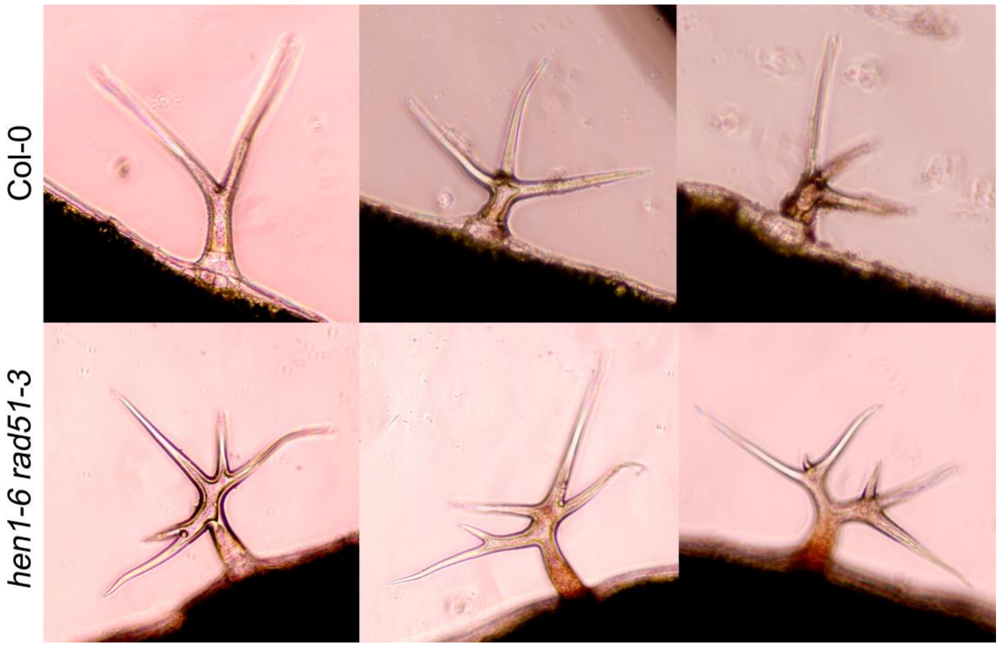

Supplementary Figure S13. Col-0 and *hen1-6 rad51-3* trichomes.

Supplementary Table S1: Mutants studied in this work.

Gene	Allele	Mutagen	Genetic background
At1g01040 (*DCL1*)	*dcl1-9*[1]	T-DNA	L*er*
	dcl1-16[6]	T-DNA	Col-0
At1g09700 (*HYL1*)	*hyl1-12*[2]	Fast neutron	L*er*
	hyl1-2[3]	T-DNA	Col-0
At4g20910 (*HEN1*)	*hen1-13*[2]	Fast neutron	L*er*
	hen1-6[3]	T-DNA	Col-0
At3g05040 (*HST*)	*hst-21*[4]	EMS	L*er*
	hst-17[6]	T-DNA	Col-0
At1g48410 (*AGO1*)	*ago1-52*[2]	EMS	L*er*
	ago1-101[5]	T-DNA	Col-0

[1-5]Supplemental References. [6]This work.

Supplementary Table S2: Percentage of decondensed nuclei at prophase I and metaphase I in PMCs from *ago1*, *dcl1*, *hen1*, *hst* and *hyl1* mutants.

Meiotic stage	Mutant	Decondensed nuclei (%)	Number of cells
Prophase I	*ago1-52*	37.74	53
	dcl1-9	40.00	20
	hen1-13	41.46	41
	hst-21	36.00	25
	hyl1-12	26.47	34
	hen1-6	34.29	35
	hyl1-2	40.00	30
Metaphase I	*hen1-6*	12.50	8
	hst-17	31.37	51
	hyl1-2	20.83	48

Supplementary Table S3: psRNATarget analysis of miRNA target candidates found among the genes up-regulated in the *ago1*, *dcl1*, *hen1*, *hst* and *hyl1* mutants.

miRNA accessibility	Target accessibility[a]	Expectation[b]	UPE	miRNA/target_aligned_fragments	Inhibition	Multiplicity[c]
ath-miR161.2	*TOPII*	3.0	19.717	`miRNA    20 UCAGUGAAAGUUACGUAACU 1`<br>`            .::: .: ::::::::::::`<br>`Target 1270 GGUCUUUGUCAAUGCAUUGA 1289`	Cleavage	1
ath-miR5021	*TOPII*	2.0	0.670	`miRNA    20 AAAAGAAGAAGAAGAAGAGU 1`<br>`            : :::::::::::::::::`<br>`Target   46 UCUUCUUCUUCUUCUUCUCC 65`	Cleavage	2
ath-miR5021	*TOPII*	2.5	0.727	`miRNA    20 AAAAGAAGAAGAAGAAGAGU 1`<br>`            : ::::::::::::::::.`<br>`Target   13 UCUUCUUCUUCUUCUUCUUC 32`	Cleavage	2
ath-miR837-5p	*SMC4*	2.0	10.579	`miRNA    20 CUUUGCUUGUUCUUUGACUA 1`<br>`            ::::: ::: ::::::::::`<br>`Target 2722 GAAACAAACCAGAAACUGAU 2741`	Translation	1
ath-miR866-5p	*SMC4*	3.0	20.510	`miRNA    21 AAUUGUUUUAGGCAAGGAACU 1`<br>`            ::.:::: : :::::.:::::`<br>`Target  590 UUGACAACAACCGUUUCUUGA 610`	Cleavage	1
ath-miR414	*SWI1*	3.0	3.376	`miRNA   20  CUGCUACUACUACUUCUACU 1`<br>`            ::.::::::::::.. ::::`<br>`Target  725 GAUGAUGAUGAUGGGAAUGA 744`	Cleavage	1
ath-miR426	*SWI1*	3.0	18.983	`miRNA    21 GCAUUCCUGUUUAAAGGUUUU 1`<br>`            :: :.: ::::::::::::.:`<br>`Target  476 CGCAGGAACAAAUUUCCAAGA 496`	Cleavage	1
ath-miR854a	*MUS81*	3.0	22.587	`miRNA    21 GAGGAGGAGGGAUAGGAGUAG 1`<br>`            .: ::.:: ::::::.:::::`<br>`Target 2012 UUGCUUCUGCCUAUUCUCAUC 2032`	Cleavage	1
ath-miR854b	*MUS81*	3.0	22.587	`miRNA    21 GAGGAGGAGGGAUAGGAGUAG 1`<br>`            .: ::.:: ::::::.:::::`<br>`Target 2012 UUGCUUCUGCCUAUUCUCAUC 2032`	Cleavage	1
ath-miR854c	*MUS81*	3.0	22.587	`miRNA    21 GAGGAGGAGGGAUAGGAGUAG 1`<br>`            .: ::.:: ::::::.:::::`<br>`Target 2012 UUGCUUCUGCCUAUUCUCAUC 2032`	Cleavage	1
ath-miR854d	*MUS81*	3.0	22.587	`miRNA    21 GAGGAGGAGGGAUAGGAGUAG 1`<br>`            .: ::.:: ::::::.:::::`<br>`Target 2012 UUGCUUCUGCCUAUUCUCAUC 203`	Cleavage	1
ath-miR854e	*MUS81*	3.0	22.587	`miRNA    21 GAGGAGGAGGGAUAGGAGUAG 1`<br>`            .: ::.:: ::::::.:::::`<br>`Target 2012 UUGCUUCUGCCUAUUCUCAUC 2032`	Cleavage	1

[a]Target accessibility - allowed maximum energy to unpair the target site (UPE) (range: 0-100, less is better): 25.0. [b]Maximum expectation (more stringent/relaxed cut-off threshold [0-2.0/4.0-5.0]): 3.0. [c]Multiplicity of target site: number of target sites for each small RNA/target pair.

Supplementary Table S4: Oligonucleotide sets used for genotyping.

Gene	Line	Oligonucleotide sequences (5´→3´)	
DCL1	*dcl1-16* (SALK_013118)	GACTTTTGGGATTCTCGAAGG	CCTCTCATCACTTTGCAAAGC
HYL1	*hyl1-2* (SALK_0064863)	AGTTCTCCCAGCGCTAATCTC	TTCTTGGAAATTGGATTGCAG
HEN1	*hen1-6* (SALK_090960)	GCATACTCCTACTCCAAAGGCGA	TGCCATCTTTCAATCCGAGCCT
HST	*hst-17* (SALK_033269)	GCAAAGCATCTCTTCTGATGGTGG	TGTCCATCACTGCTACATGCTGAG
AGO1	*ago1-101* (SALK_035319)	GGCAATCCAGCCTATACCTTC	TTCCTCGATGAGTCACTTCAAC
RAD51	*rad51-3* (SAIL_873_C08)	TCTCAAGAACTTTGCAAGATGC	ATGCCAAGGTTGACAAGATTG

Supplementary Table S5: Antibodies used for immunolocalization.

Antibody	Dilution	Obtained in
anti-ASY1[1]	1:1000	Rat
anti-ZYP1[1]	1:250	Rabbit
anti-DMC1[1]	1:300	Rabbit
anti-H3K9me2[2]	1:200	Rabbit
anti-H3K4me2[3]	1:200	Rabbit
anti-H3K4me3[3]	1:200	Rabbit
anti-H3K27me3[2]	1:200	Rabbit
anti-H3Ac[2]	1:200	Rabbit
anti-H3S10Ph[4]	1:200	Rabbit

[1]Kindly donated by Prof. Chris Franklin, University of Birmingham, UK; [2]Millipore; [3]Abcam; [4]Upstate.

Supplementary Table S6: Oligonucleotide sets used for RT-qPCR.

Gene		Oligonucleotide sequences (5´→3´)		UPL	Amplification product (bp)
Symbol	AGI code	Forward primer	Reverse primer		
SYN1	At5g05490	TCAGCAGACTTTTATTTCCATGC	TCAGCTTGATGGAACTGCTG	129	93
SMC1	At3g54670	GCTCGAAGCTTGGAAAAATTC	CCACGTCCTTTCTATTGGTCTC	63	96
SMC6A	At5g07660	TGCCTCAAGATGCAACAAAC	AAAGTCGAGAAAGACCGTTCC	150	76
SMC6B	At5g61460	TCGCACGAGAGGATAAAGAAA	TGACTCAAAGCCGAGGATG	68	106
TOPII	At3g23890	CCTATTCAGCAATGGACACAGA	TTCTTCTTTGCTCCTTGTCTACC	139	76
SMC4A	At5g48600	TGAAGGATAAACTGCAAACCAA	TGATCTCTGTATTGTTTTTGTCGAT	131	114
CHR11	At3g06400	AGCCAGACTTAAAGAGATGCAGA	CTCCCTTTTCCCTTATTGTTCA	67	108
MET1	At5g49160	CAACTGCAATGTGATTCTTAGGG	GCCTCTGTAGTAGAGACACAATCATC	39	75
SWI1	At5g51330	TTATTGGAGAAACCTTGCGTAAA	CCACTGTTTTCTTAAGCCATCC	69	60
SPO11-1	At3g13170	TTCCCAAACAGTGTCTTTTGC	TTCAAGTTCCAACCTCCATTG	143	113
ATM	At3g48190	AGGGTGGTGAGATGAGAAGC	TCTGTGTCAATTGCGTCTTGT	98	67
ATR	At5g40820	TTCAGCGCCCAAAGAAGA	GGCTTGCAGAGGAATGGATA	3	67
BRCA1	At4g21070	CCAAGAAATTGGTCTTATCTTGC	AGTTCCGCAAATTCTGCAAT	100	73
BRCA2B	At5g01630	CACCTTAAAACCCGCAGTG	AGGTGATTTACAAGCACCGATT	140	117
RAD50	At2g31970	GCAGTGCAGGTCAAAAGGTT	GGCCCATCCAGGTTTGTAG	136	118
RAD51C	At2g45280	TCAACTAGCGCTTGCTTTAGG	AATACAGAATGACTCGGTTGGTG	54	65
RAD51	At5g20850	CATGCCACCACAACAAGG	ACATGGCGAGCTTATCACTTTAC	91	78
DMC1	At3g22880	TCAACGTTGCTGTCTACATGACT	GACCACCTGCTGGCTTTTT	31	90
MSH4	At4g17380	CAAGAATGGGGACAATGGAT	TGCATTATGAAAGCGGTCTCT	151	82
MLH3	At4g35520	GACTGAAGCAGACCTCACTTTG	GCCTTCAAATCGACAAGAGG	47	76
MUS81	At4g30870	GATATGTACCCAACGCTTTTGTC	CTTCTTGCGCCGAGACAT	29	73
FANCM	At1g35530	GCGGTAAAAAGCACATTAGAGG	GCTCCCGAAGAGACCTCAG	153	94

UPL: Universal Probe Library.

Supplementary References

1. Jacobsen, S. E., Running, M. P. & Meyerowitz, E. M. Disruption of an RNA helicase/RNAse III gene in Arabidopsis causes unregulated cell division in floral meristems. *Development* **126**, 5231-5243 (1999).
2. Jover-Gil, S., Candela, H. & Ponce, M.R. Plant microRNAs and development. *Int. J. Dev. Biol.* **49**, 733-744 (2005).
3. Vazquez, F., Gasciolli, V., Crété, P. & Vaucheret, H. The nuclear dsRNA binding protein HYL1 is required for microRNA accumulation and plant development, but not posttranscriptional transgene silencing. *Curr. Biol.* **14**, 346-351 (2004).
4. Berná, G., Robles, P. & Micol, J.L. A mutational analysis of leaf morphogenesis in *Arabidopsis thaliana*. *Genetics* **152**, 729-742 (1999).
5. Miyashima, S., Hashimoto, T. & Nakajima, K. ARGONAUTE1 acts in Arabidopsis root radial pattern formation independently of the SHR/SCR pathway. *Plant Cell Physiol.* **50**, 626-634 (2009).
